# Soluble tissue factor generated by necroptosis-triggered shedding is responsible for thrombosis

**DOI:** 10.1038/s41422-025-01167-8

**Published:** 2025-09-12

**Authors:** Peixing Wan, Swati Choksi, Yeon-Ji Park, Xin Chen, Jiong Yan, Sahar Foroutannejad, Zhaoshan Liu, Jichun Chen, Ross Lake, Chengyu Liu, Zheng-Gang Liu

**Affiliations:** 1 National Cancer Institute; National Institutes of Health, Laboratory of Cellular and Molecular Biology, Bethesda, MD USA; 2 National Heart, Lung, and Blood Institute; National Institutes of Health, Transgenic Core, Bethesda, MD USA; 3 National Cancer Institute; National Institutes of Health, Laboratory of Cancer Biology and Genetics, Bethesda, MD USA

**Keywords:** Necroptosis, Immunology

## Abstract

Tissue factor (TF) is a cell surface protein critical for normal hemostasis and pathological thrombosis. Necroptosis is a form of regulated necrosis associated with different diseases. Here, we reported the identification of the first functional soluble tissue factor (sTF) in mediating blood coagulation, shed from the membrane full-length TF (flTF) by proteases, ADAMs, during necroptosis. By generating sTF-specific antibody and transgenic mice carrying knockin mutations at the ADAM cleavage site of TF (T211V212 mutated to E211E212), we demonstrated that this sTF is responsible for necroptosis-related thrombosis in inflammation and viral infection mouse models. Importantly, we showed that eliminating necroptosis or the cleavage of the flTF blocked the production of sTF and prevented thrombosis in mice. We also detected sTF in the plasma of human COVID-19 patients and showed that SARS-CoV-2 pseudovirus induced sTF production. Our findings demonstrated that the sTF plays a major role in thrombosis under necroptosis-related pathological conditions and provided a diagnostic marker and potential therapies for treating thrombosis without affecting hemostasis.

## Introduction

Tissue factor (TF) is the main trigger of blood coagulation and is essential for normal hemostasis and pathological thrombosis.^1,2^ TF is predominantly expressed in subendothelial tissues under physiological conditions and remains segregated from the bloodstream,^3^ ensuring a tightly regulated hemostatic response. However, upon vascular injury or perturbations, it becomes exposed and readily activates the circulating FVII to initiate the blood clotting cascade.^4^ TF expression could also be potently induced in monocytes and macrophages under pathological conditions^5^ by endotoxin and inflammatory cytokines such as lipopolysaccharide,^6^ TNF-α^7^ and interleukin-1.^8^ As TF is a glycosylated membrane protein and its interaction with phosphatidylserine (PS) is known to be critical for TF coagulation activity,^9,10^ the cell surface localized and extracellular microvesicles (MVs)-associated full-length TF (flTF) is believed to be responsible for mediating blood coagulation under both physiological and pathological conditions.^11,12^ Although an alternatively spliced TF (asTF) is produced as a soluble TF variant, the asTF was found to have no activity in mediating blood coagulation.^13^ In addition, early studies reported that the ectodomain of TF (EctoTF) has limited coagulation activity compared to flTF.^14,15^ Thrombosis is a pathological process that leads to abnormal blood clots and results in clinical consequences. Under various pathological conditions like inflammation and viral infection, elevated plasma levels of TF were observed, leading to diffused thrombosis which can result in microvascular occlusion and organ dysfunction.^16,17^ It is also reported that TF on monocyte/macrophage plays a critical role in initiating thrombosis in those conditions.^18,19^ Although recent reports hinted at the existence of soluble TF proteins under certain pathological conditions, the origin of these soluble TF proteins and their thrombogenic properties remain elusive.^20^

Necroptosis is a form of programmed necrotic cell death, originally discovered as an alternative cell death when apoptosis is blocked after the engagement of death receptors.^21,22^ The canonical death receptor-mediated necroptotic pathway composed of RIPK1-RIPK3-MLKL is activated downstream of death receptors (e.g., TNFR and Fas).^23^ Phosphorylation of MLKL by RIPK3 leads to the oligomerization and the plasma membrane translocation of the protein and the subsequent permeabilization of plasma membranes and organelles, followed by uncontrolled release of intracellular material, which elicits immune responses.^24^ The translocation of MLKL to the plasma membrane also results in the activation of ADAM proteases and the shedding of cell surface proteins.^25^ The immune system has also evolved ways to eliminate viral infection by activating RIPK1-independent RIPK3-MLKL pathway. RHIM-domain-containing proteins such as ZBP1 and TRIF can bind directly to RIPK3 through its RHIM domain, resulting in the activation of RIPK3-MLKL pathway and necroptosis.^26^

Necroptosis has been implicated in many pathological conditions including inflammation and viral infection.^27^ Necroptosis is known to contribute in the development of multiple inflammation-related diseases such as cancer,^28^ while it is also well established that necroptosis is a viral defense mechanism to restrict virus replication.^29^ Recent reports suggest that necroptosis may play a role in promoting thrombosis under some pathological conditions.^30^ While thrombosis is a clinical outcome of necroptosis-induced inflammation and viral infection, and necroptosis represents a prominent form of cell death triggered by inflammation and viral infection, the role of necroptosis in the process of thrombosis is still elusive, and the underlying mechanism of thrombosis under these conditions is unclear. For example, thrombosis is reported as one of the severe symptoms in COVID-19 patients^31^ and necroptosis is detected in SARS-CoV-2-infected human tissues.^32,33^ However, it is not clear whether necroptosis contributes to thrombosis in response to SARS-CoV-2 virus infection in COVID-19 patients.

In our current study, we reported that a truncated TF is generated from the shedding of the monocyte/macrophage membrane flTF by cell surface proteases, ADAMs, during necroptosis. The cleavage of flTF at the site of T211V212 by ADAMs yields the TF1-211, which is designated as sTF in our study. Importantly, we found that the sTF, in contrast to both the EctoTF (TF1-223) and the asTF, is fully functional in mediating coagulation, although its activity is slightly lower than that of the membrane flTF. We also found that while EctoTF and asTF do not bind to PS, sTF and flTF bind to PS similarly and the binding of sTF to PS is critical for its activity. By generating a sTF-specific antibody and a mouse strain with mutations in the ADAM cleavage site of flTF, we demonstrated that the sTF, not the MVs-associated flTF, plays a major role in thrombosis in two preclinical animal models. We also showed that the sTF was detected in blood samples of COVID-19 patients. Additionally, SARS-CoV-2 pseudovirus triggered necroptosis in monocytes/macrophages and induced the shedding of flTF to produce sTF. Our findings elucidate the crucial mechanism driving thrombosis in the context of necroptosis-induced inflammation and viral infection. They also offer a valuable marker for the early diagnosis of thrombosis and highlight potential targeted therapies for treating thrombosis caused by necroptosis-induced inflammation or viral infection without affecting normal hemostasis.

## Results

### Necroptosis is crucial for thrombosis in mouse inflammation model

**Fig. 1:**
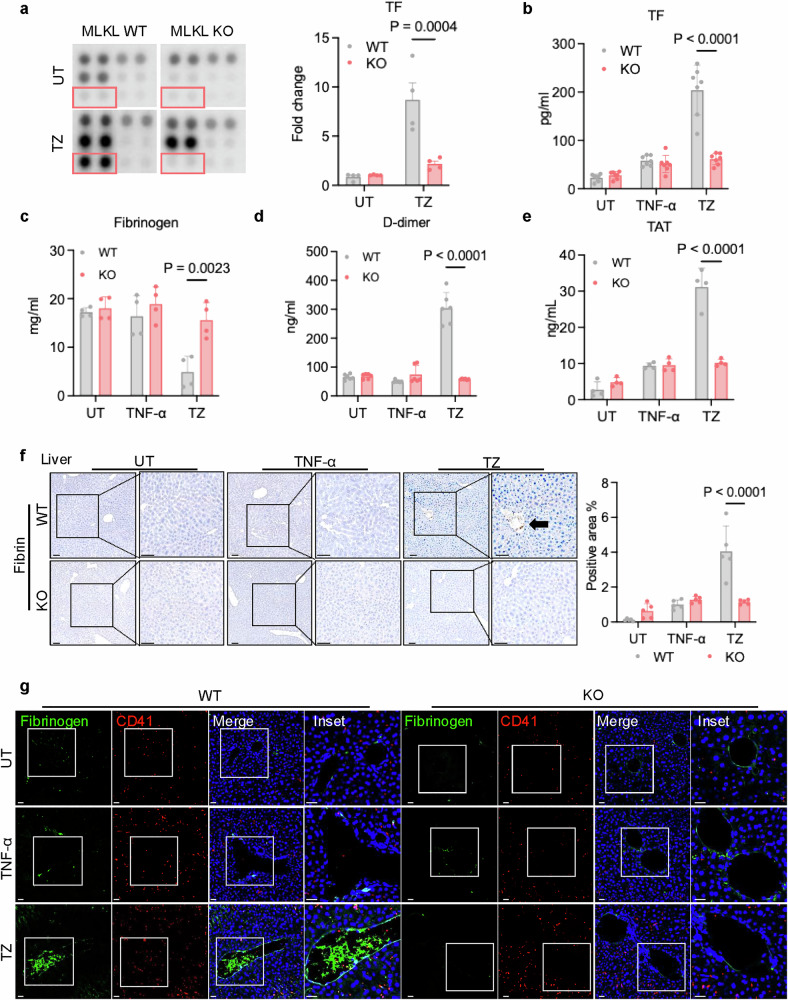
Necroptosis is crucial for thrombosis in mouse inflammation model. WT and MLKL KO mice received ZVAD (i.p.) 15 min before and 1 h after the TNF-α (i.v.) injection to induce necroptosis-driven inflammation. All the assays were done at 6 h post TNF-α delivery unless specified. **a** Plasma samples from untreated (UT) and TZ-treated WT and MLKL KO mice were examined with cytokine array. Representative image of the cytokine array is shown here. TF spots were highlighted in red box. Quantification of TF level on arrays is shown in right panel. **b** Measuring TF level in mouse plasma from UT, TNF-α-, or TZ-challenged WT and MLKL KO mice by ELISA assay. *n* = 7 per group. **c**–**e** Measuring classical markers of thrombosis, Fibrinogen, D-dimer, and TAT by ELISA assays. Plasma samples from UT, TNF-α, or TZ-challenged WT and MLKL KO mice were examined. *n* = 4 per in Fibrinogen and TAT assays, *n* = 6 in D-dimer assay. **f** Representative images of fibrin immunohistochemistry (IHC, left panel) of liver sections from untreated, TNF-α-, or TZ-challenged WT and MLKL KO mice at 6 h post treatment. Right panel, fibrin IHC staining quantification is shown here. Scale bars = 40 µm. Arrow: fibrin signal. **g** Representative immunofluorescence (IF) images of liver sections from UT, TNF-α-, or TZ-challenged WT and MLKL KO mice are shown. Fibrinogen deposition was indicated by a green signal, platelets were labeled with CD41 (red), and nuclei were stained blue. Scale bars = 40 µm.

As TNF-α and z-VAD-FMK (TZ)-induced necroptosis is critical for the inflammatory response, the mouse model with TZ treatment is well established as a murine model of necroptosis-induced inflammation (Supplementary information, Fig. S1a).^27,34,35^ We observed tissue damage in wild-type (WT) mice challenged with TZ but not in necroptosis-deficient MLKL knockout (KO) mice (Supplementary information, Fig. S1b). WT mice had a significant drop in body temperature and increased mortality post TZ treatment, whereas MLKL KO mice were not affected (Supplementary information, Fig. S1c, d). TNF-α alone had no effect on tissue damage, and thus it was utilized as a control in the study. Using mouse cytokine array, we examined the changes of cytokine profiles in these mice and observed significant elevated expression levels of various proteins in the plasma of TZ-treated WT but not MLKL KO mice (Supplementary information, Fig. S2a‒d). Particularly, we found that TF levels were increased by > 5-fold in TZ-challenged WT mice compared to untreated controls, peaking at 6 h after TZ treatment (Fig. 1a; Supplementary information, Fig. S2e), which was confirmed by ELISA (Fig. 1b). Interestingly, MLKL KO mice challenged with TZ showed no change in plasma TF levels (Fig. 1a, b). TNF-α treatment alone also increased plasma TF by 2-fold but exhibited no difference between WT and MLKL KO mice (Fig. 1b).

TF is known as the main trigger of blood coagulation and is crucial for hemostasis and thrombosis. In the TZ model, we found significant hemolysis, reduced clotting time, and slightly lower platelet percentage in TZ-treated WT mice but not in TZ-treated MLKL KO mice (Supplementary information, Fig. S2f‒h). The classical markers of thrombosis were examined at different time points post TZ challenge (Supplementary information, Fig. S2i‒k). A decrease in fibrinogen levels was seen, while the D-dimer and thrombin-antithrombin complex (TAT) levels were elevated in WT but not in MLKL KO plasma samples at 6 h post TZ treatment (Fig. 1c–e). Disseminated thrombus formation was observed in liver and kidney sections from WT but not MLKL KO mice (Fig. 1f, g; Supplementary information, Fig. S3a, b). These results suggest that necroptosis may play a key role in TZ-induced thrombosis. The critical role of necroptosis in thrombosis was confirmed by inhibiting necroptosis with the RIPK1-specific inhibitor, necrostatin-1 (Nec-1) (Supplementary information, Fig. S4a‒f). The inhibition of necroptosis by Nec-1 was confirmed by lactate dehydrogenase (LDH) assay (Supplementary information, Fig. S4g). Taken together, our results indicate that necroptosis may play a key role in triggering inflammation-related thrombosis.

### Soluble TF mediates necroptosis-dependent hypercoagulation

**Fig. 2:**
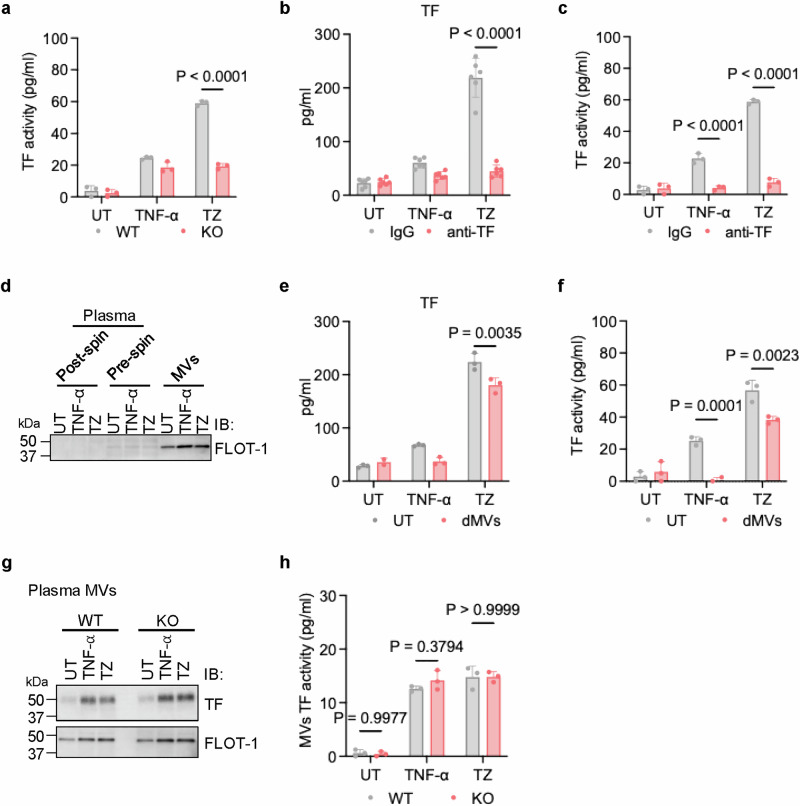
Soluble TF mainly mediated necroptosis-dependent hypercoagulation. **a** Plasma samples from UT, TNF-α-, or TZ-challenged WT and MLKL KO mice were examined for TF activity by PCA assay. *n* = 3 per group. **b**, **c** TF was selectively depleted from the plasma samples of UT, TNF-α-, or TZ-challenged WT mice using an antibody pull-down technique. **b** The TF levels in the plasma pre-processed with either control IgG or anti-TF antibody was measured by ELISA. *n* = 6 per group. **c** The TF activity was measured in either control IgG or anti-TF antibody pre-processed plasma by PCA assay. *n* = 3 per group. **d**–**h** MVs were isolated from plasma of UT, TNF-α- or TZ-challenged WT mice by spin down at 100,000× *g* for 20 min. **d** Post-spin plasma fraction (MV-depleted plasma), pre-spin plasma samples, and isolated MVs were checked by western blot for Flotillin-1 (FLOT-1). **e** The TF levels in plasma and MV-depleted plasma fraction was measured by ELISA. *n* = 3 per group. **f** The TF activity was measured in plasma and MV-depleted plasma fraction by PCA assay. *n* = 3 per group. dMVs: depleted MVs. **g** MVs isolated from plasma of UT, TNF-α or TZ-challenged WT and MLKL KO mice were examined by western blot with the indicated antibodies. **h** The TF activity was measured in MVs isolated from plasma of UT, TNF-α- or TZ-challenged WT and MLKL KO mice by PCA assay. *n* = 3 per group.

We next measured the coagulation activities of these samples by the procoagulant assay (PCA), which detects the TF-dependent conversion of FX to FXa.^12,36^ TNF-α modestly induced TF activity in both WT and MLKL KO plasma samples. However, a substantial increase in the plasma TF activity was detected in TZ-challenged WT mice, but such inductions were not observed in MLKL KO samples (Fig. 2a). When a monoclonal anti-TF antibody was used to deplete TF (Fig. 2b), TZ-elevated TF activity was abolished (Fig. 2c), suggesting that the necroptosis-triggered hypercoagulation may be dependent on TF in plasma. The efficiency of TF depletion by the anti-TF antibody was confirmed in later experiments after determining the various forms of plasma TFs (Fig. 6a). Studies have suggested that TF can be released from cells in the form of MVs.^12^ We assessed the role of MV-associated TF in hypercoagulation by removing MVs from plasma samples. After MVs removal, TF levels returned to basal ones in the plasma of TNF-α-treated mice and partially decreased in the plasma of TZ-treated mice but still remained highly elevated (Fig. 2d, e), suggesting the possible presence of MV-independent soluble form of TF (sTF).

Importantly, removing MVs reduced TF procoagulant activity to basal levels in the plasma of TNF-α mice but only reduced by about 28% in the plasma of TZ-treated mice (Fig. 2f), suggesting the possibility that MV-associated flTF has a minor role while MV-independent sTF plays a major role in the hypercoagulation of TZ-treated plasma. Levels of MV-associated TF were significantly increased following TNF-α or TZ treatment in both WT and MLKL KO mice. It is likely that both the elevated TF expression and MVs secretion contributed to this increase (Fig. 2g). The activity of MV-associated TF is comparable between TNF-α- and TZ-treated groups of both WT and MLKL KO mice (Fig. 2h), significantly lower than the coagulation activity in MV-depleted plasma of TZ-treated WT mice (Fig. 2f). In summary, our results suggest the possibility that MV-independent sTF, rather than MV-associated flTF, is the major contributor of TZ-induced hypercoagulation.

### Necroptosis prompts the release of sTF from monocytes/macrophages

**Fig. 3:**
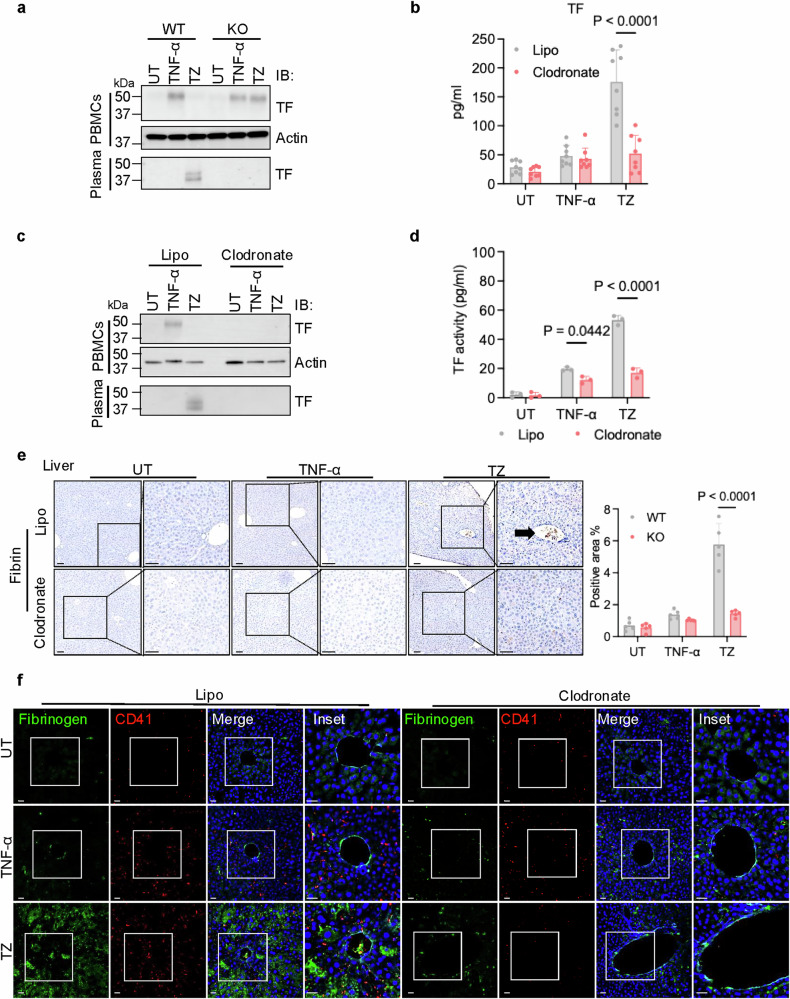
Necroptosis prompts the release of sTF from monocytes/macrophages in vivo. **a** Mouse PBMCs and plasma from UT, TNF-α-, or TZ-challenged WT and MLKL KO mice were examined by western blot with the indicated antibodies. MVs were removed from plasma samples. **b** Plasma TF levels in WT mice pre-treated with either control liposome (Lipo) or clodronate and challenged with UT, TNF-α, or TZ were measured by ELISA. *n* = 8 per group. **c** Mouse PBMCs and plasma samples from WT mice pre-treated with either control liposome (Lipo) or clodronate and challenged with UT, TNF-α, or TZ were examined by western blot with the indicated antibodies. MVs were removed from plasma samples. **d** Assessing TF activity in plasma samples from WT mice pre-treated with either control liposome (Lipo) or clodronate and challenged with UT, TNF-α, or TZ by PCA assay. *n* = 3 per group. **e** Liver sections from WT mice pre-treated with either control liposome (Lipo) or clodronate and challenged with UT, TNF-α, or TZ were examined. Representative images of fibrin IHC staining are shown in left panel. Right panel, fibrin IHC staining quantification is shown. Scale bars = 40 µm. Arrow: fibrin signal. **f** Liver sections from WT mice pre-treated with either control liposome (Lipo) or clodronate and challenged with UT, TNF-α, or TZ were examined. Representative images of IF staining are shown. Fibrinogen deposition was indicated by a green signal, platelets were labeled with CD41 (red), and nuclei were stained blue. Scale bars = 40 µm.

As TF levels on monocytes/macrophages could be potently induced under inflammatory conditions,^5^ we investigated whether the plasma TF comes from monocytes/macrophages. We first examined the expression of TF in plasma and peripheral blood mononuclear cells (PBMCs) from WT and MLKL KO mice. The specificity of this anti-TF antibody was validated with the ectopically expressed, HA-tagged mouse flTF protein and was further confirmed in later experiments (Supplementary information, Fig. S5a). TNF-α elevated TF expression in PBMCs in both WT and MLKL KO mice, whereas elevated TF levels were only observed in MLKL KO, but not WT PBMCs isolated from TZ-treated mice (Fig. 3a, top panel). Meanwhile, sTF was observed only in plasma of TZ-treated WT, not MLKL KO mice and displayed a molecular weight close to 37 kDa (Fig. 3a, bottom panel), which is smaller than the ~52 kDa flTF detected in PBMCs. These results suggested that the plasma sTF may be produced from monocytes/macrophages of WT mice following TZ treatment. To examine this possibility, monocytes/macrophages were selectively eliminated in mice by clodronate liposome. Post monocyte/macrophage depletion, plasma TF levels in TNF-α-treated mice remained similar to controls, while TZ-challenged mice had significantly lower plasma TF levels than control liposome-treated mice (Fig. 3b). In clodronate-treated mice, the TNF-α-induced flTF expression in PBMCs and the TZ-induced smaller sTF in plasma were not detected (Fig. 3c). Also, clodronate abolished TZ-elevated TF activity in mouse plasma (Fig. 3d). The TZ-induced hemolysis and the rapid blood clotting were reversed by clodronate (Supplementary information, Fig. S5b, c). Plasma levels of fibrinogen, D-dimer, and TAT remained unchanged following clodronate administration in both control- and TNF-α-treated mice. However, clodronate reversed the TZ-induced alterations in these levels (Supplementary information, Fig. S5d‒f). Clot formation observed in the livers and kidneys of TZ-treated mice was dramatically reduced when monocytes/macrophages were depleted by clodronate (Fig. 3e, f; Supplementary information, Fig. S6a, b). These data suggest that monocyte/macrophages are critical for producing plasma sTF and TZ-induced hypercoagulation. To further support that sTF is primarily produced from monocytes/macrophages, we used *TF*^*fl*/*fl*^ and *LysM*-*Cre* mice from the Jackson Lab to generate *LysM-Cre*;*TF*^*fl*/*fl*^ mice for evaluating sTF production with TZ treatment. While sTF is produced in *TF*^*fl*/*fl*^ mice treated with TZ, it could not be detected in TZ-treated *LysM*-*Cre*;*TF*^*fl*/*fl*^ mice. No TF coagulation activity was detected in the plasma samples from these TZ-treated *LysM*-*Cre*;*TF*^*fl*/*fl*^ mice (Supplementary information, Fig. S5g‒i). These results confirmed that sTF is primarily produced from monocytes/macrophages.

To evaluate the production of sTF from monocytes/macrophages, we examined TF expression in J774A.1 cells, a murine monocytic cell line. A substantial increase in TF expression upon TNF-α stimulation was observed, but not in cell lysate when necroptosis (indicated by pMLKL) was triggered by TZ treatment (Supplementary information, Fig. S7a). IF imaging showed that TNF-α-induced TF mainly resided on the plasma membrane of the cells, which was largely diminished following TZ treatment (Supplementary information, Fig. S7b). When conditioned media (CM) were examined, we detected an sTF band in CM of TZ-treated J774A.1 cells with the same size as the one seen in the plasma of TZ-treated mice (Fig. 3a; Supplementary information, Fig. S7a, bottom panel). This sTF was not seen in CM of cells treated with TNF-α or ZVAD alone. To rule out the possibility that this smaller sTF present in the CM is the unmodified flTF leaked from dying cells, we examined the mobilities of flTF and sTF after de-glycosylation by PNGase. As shown in Supplementary information, Fig. S7c, both the flTF and sTF are glycosylated. And the de-glycosylated flTF from cell lysate is clearly distinct from the sTF seen in CM, indicating that sTF is not the unmodified flTF. As sTF is primarily produced from monocytes/macrophages by the cleavage of the glycosylated flTF, the two bands of sTF seen in blot represented different glycosylation forms.

We then assessed the coagulation activity of CM and found that only CM from TZ-treated J774A.1 cells displayed significantly elevated TF activity (Supplementary information, Fig. S7d). Using the anti-TF antibody to deplete TF from this CM completely abolished its coagulation activity (Supplementary information, Fig. S7d). Furthermore, removing MVs almost eliminated the TF from TNF-α-treated CM (Supplementary information, Fig. S7e), but only resulted in a minor decrease of TF activity in CM from TZ-treated J774A.1 cells (Supplementary information, Fig. S7f). Additionally, the removal of MV-associated flTF completely abolished the procoagulant activity in CM from TNF-α-treated cells, but only partially reduced the activity in CM from TZ-treated cells (Supplementary information, Fig. S7f).

**Fig. 4:**
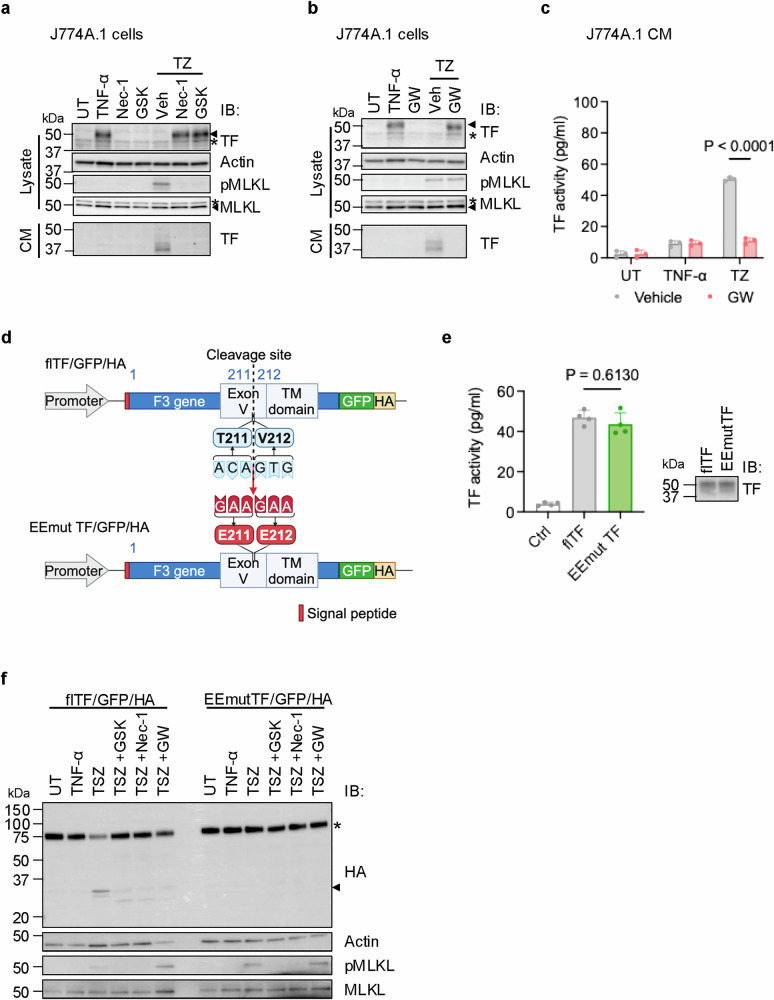
The sTF is shed from necroptotic cell surface by ADAMs. **a** J774A.1 cells were treated with necroptosis inhibitors, Nec-1 and GSK, 1 h before TZ treatment. J774A.1 cells were then treated with TZ for a duration of 6 h. Cell lysate and CM from treated J774A.1 cells were examined by western blot with the indicated antibodies. MVs were removed from CM. Arrowhead: target bands. Star: unspecific bands. **b** Western blot analysis was performed on J774A.1 cells that were either UT, treated with TNF-α alone, treated with GW280264X (GW) alone, treated with TZ, or pre-treated with GW for 1 h before TZ treatment, all for a duration of 6 h. Cell lysates and CM were immunoblotted with the indicated antibodies. MVs were removed from CM. Arrowhead: target bands. Star: unspecific bands. **c** The TF activity was measured in CM from vehicle or GW pre-treated J774A.1 cells that were either UT, treated with TNF-α or TZ by PCA assay. *n* = 3 per group. **d** Schematic diagram showing T211V212 as the potential cleavage site in flTF/GFP/HA. E211E212-mutated TF (EEmutTF-GFP-HA) was generated to block the cleavage. **e** Same amount of recombinant flTF or EEmutTF protein was purified from HEK293 cells as detected by western blot (right panel). Empty vector was used as control. These relipidated TF proteins were examined by PCA analysis for procoagulant activity (left panel). *n* = 4 per group. **f** Due to the low transfection efficiency of J774A.1 cells, MEF cells were transfected with either flTF/GFP/HA or EEmutTF/GFP/HA plasmids. After 8 h, cells were treated with Nec-1, GSK, or GW for 1 h, followed by TNF-α + Smac mimetic + ZVAD (TSZ) to induce necroptosis. Cell lysates were examined 16 h after the TSZ treatment by western blot with the indicated antibodies. Arrowhead: cleaved protein. Star: full-length protein.

We next evaluated the role of necroptosis in the production of this sTF and found that necroptosis inhibitors, Nec-1 and GSK’872 (GSK), blocked the production of sTF in CM and restored the level of flTF in cell lysate of TZ-treated J774A.1 cells (Fig. 4a). We obtained similar results with bone marrow derived macrophages (BMDMs) isolated from WT mice (Supplementary information, Fig. S7g, upper panel). MLKL KO BMDMs are resistant to necroptosis-triggered flTF reduction in cell lysate (Supplementary information, Fig. S7g, lower panel). The high basal levels of TF in non-treated BMDMs are most likely due to the accumulation of autocrine inflammatory cytokines in the culture medium during the extended in vitro differentiation of these cells. The comparison of the flTF levels in PBMCs, BMDMs and J774A.1 cell lysate and the sTF levels in mouse plasma, CMs of BMDMs and CMs of J774A.1 cells were shown in Supplementary information, Fig. S7h. Taken together, these results suggest that necroptosis induces the release of sTF from monocytes/macrophages and the sTF may play a major role in hypercoagulation.

### The sTF is shed from cell surface by ADAMs

As we reported previously, the necroptotic signal leads to the activation of cell surface proteases, ADAMs.^25^ The concurrent decrease of flTF on cell surface and the increase of sTF in CM suggests that the sTF might be generated through shedding of the cell surface flTF from monocytes/macrophages by ADAMs under necroptotic conditions. As ADAM10/17, present in both mouse and human monocytes/macrophages,^37^ were the pivotal players in cell surface protein shedding during necroptosis,^21^ we employed the ADAM10/17 inhibitor, GW 280264X (GW) to discern this possibility. In TZ-treated J774A.1 cells, GW production retained the level of flTF in the lysate and blocked the appearance of sTF in CM (Fig. 4b), indicating that ADAMs are likely responsible for producing the sTF. Administration of GW also markedly abolished the procoagulant activity of the CM from TZ-treated J774A.1 cells (Fig. 4c). The inhibitory effects of GW on the sTF production and TF activity in CM was further confirmed in human primary macrophages and U937 cells (Supplementary information, Fig. S8a‒c). These results suggest that the sTF is shed from the surface of necroptotic monocytes/macrophages by ADAMs.

To identify the ADAM10/17 cleavage site of flTF, we used the MEROPS system^38^ to search for possible sites, and subsequently mutated the most likely potential cleavage site by changing T211V212 into two Es (EEmutTF, Fig. 4d). We confirmed that the EE mutation did not alter the cell surface localization of TF (Supplementary information, Fig. S8d). The procoagulant activity of purified flTF and EEmutTF proteins was comparable when tested in the in vitro coagulation activity assays (Fig. 4e). WT TF was cleaved during TSZ-induced necroptosis as the cleaved C-terminal fragment of flTF was detected in cell lysate and this cleavage was blocked by necroptosis inhibitors or GW (Fig. 4f). However, the EEmutTF protein remained intact, and no cleaved C-terminal fragment was detected in necroptotic cells (Fig. 4f). These results confirmed that T211V212 is the ADAM10/17 cleavage site of flTF and this cleavage yields the sTF, TF1‒211.

### The sTF, but not EctoTF, is functional at mediating coagulation

**Fig. 5:**
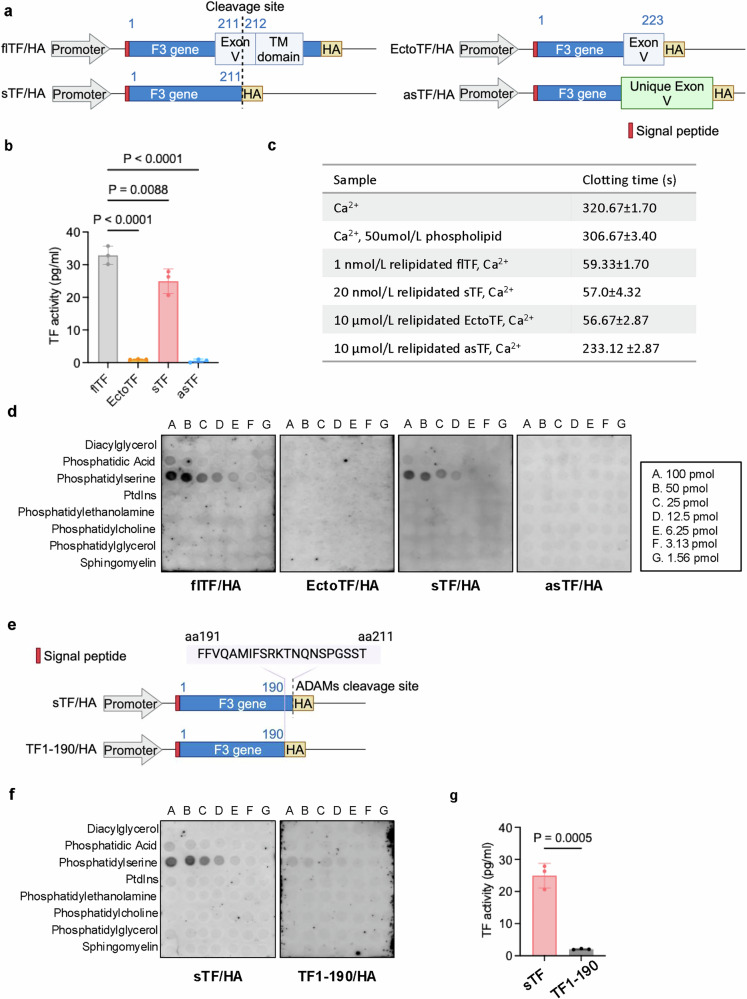
The sTF has similar coagulation activity to the flTF. **a** Schematic diagram of HA-tagged plasmids of flTF, EctoTF, the sTF generated by ADAM cleavage (TF aa1‒211), and the asTF. **b** Recombinant flTF, EctoTF, sTF, or asTF protein was purified from HEK293 cells and relipidated (Supplementary information, Fig. S8e). These relipidated TF proteins were examined by PCA analysis for procoagulant activity. *n* = 3 per group. **c** Recombinant mouse TF proteins, including flTF, EctoTF, sTF, and asTF protein, were purified from HEK293 cells and relipidated. Clotting time of the pooled normal mouse plasma in the presence of relipidated TF proteins was determined in the PT assay by the semi-automatic coagulation analyzer. *n* = 3 per group. **d** Recombinant mouse TF proteins, including flTF, EctoTF, sTF, and asTF protein, were purified from HEK293 cells. Lipid binding affinity of these TF proteins were determined by protein-lipid overlay assay using Membrane Lipid Strips. Representative images are shown here. **e** Schematic diagram showing C-terminal tail of sTF. TF1‒190/HA was generated by removing the C-terminal tail of sTF. **f**, **g** Recombinant mouse TF1‒190/HA and sTF/HA proteins were purified from HEK293 cells. Lipid binding affinity of TF1‒190/HA or sTF/HA was determined by protein-lipid overlay assay using Membrane Lipid Strips. Representative images are shown here (**f**). TF1‒190/HA or sTF/HA protein was relipidated and examined by PCA analysis for procoagulant activity (**g**). *n* = 3 per group.

As the procoagulant activity of TF was attributed to the membrane flTF and it is known that the ectodomain of TF, TF1‒223 (EctoTF),^14,15^ and the asTF^13,39^ have limited/no coagulation activity, and our data from plasma and CM samples suggested that the sTF, which is different from Ecto TF by missing the last 12 amino acids of EctoTF C-terminus, has high procoagulant activity, we decided to verify our findings by directly comparing the procoagulant activities of flTF, EctoTF, sTF, and asTF proteins by PCA. To do so, we constructed mammalian expression plasmids encoding the flTF, EctoTF (amino acids 1‒223), sTF (amino acids 1‒211) and asTF (Fig. 5a; Supplementary information, Fig. S8e) and relipidated the purified different TF proteins for evaluating their procoagulant activities. As shown in Fig. 5b and Supplementary information, Fig. S8f, flTF and sTF have high procoagulant activities, although sTF activity is slightly lower than the flTF activity. In contrast, both EctoTF and asTF showed minimal activity, which is consistent with previous reports.^13^ To further confirm that sTF, but not EctoTF, has significant coagulation activity, we used the prothrombin time (PT) assay to evaluate TF-dependent clotting times in normal plasma and compare the coagulation activities of the different TF proteins.^40,41^ The results of these PT assays showed again that sTF has much higher coagulation activity than the EctoTF does, as about 500-fold more of EctoTF is needed for triggering the clotting of normal plasma within the similar time, although flTF has higher activity than sTF does (Fig. 5c). Therefore, these results confirmed that the sTF, generated from shedding of the membrane flTF, has much higher coagulation activity although its activity is slightly lower than that of flTF. Also, as the binding to phospholipids, particularly PS is critical for TF function,^34,35^ these results of PT assay suggest that the phospholipid existing in the normal plasms is sufficient for sTF to be functional without any blood cells. To investigate whether blood cells including platelets contribute to the elevation of sTF coagulation activity, we tested whether platelets or PBMCs will have the same effect on sTF activity as PS does. We found that while the presence of PS leads to the dramatic elevation of sTF coagulation activity, platelets and PBMCs failed to do so (Supplementary information, Fig. S8g). Taken together, all these data suggest that blood cells do not play a major role in the direct modulation of sTF coagulation activity.

To investigate the possible mechanism underlying the procoagulant activity of sTF, we examined the lipid binding abilities of these different TF proteins by lipid‒protein interaction assay. As shown in Fig. 5d, only flTF and sTF, not EctoTF and asTF, bind to PS with similar affinity. These results implicated that the high binding affinity of sTF to PS may be critical for its procoagulant activity. As there is a disulfide bond (Cys190‒Cys213) at the C-terminus of EctoTF, the cleavage at T211‒V212 disrupted this disulfide bond and generates a different C-terminal end in sTF. As the last 21 amino acids (F191FVQAMIFSRKTNQNSPGSS-T211) of sTF C-terminus includes a stretch of hydrophobic residues (FFVQAMIF), a positively charged cluster (RK), and a region rich in asparagine, serine, and glutamine, all of which could collectively promote and stabilize sTF’s interaction with PS, we tested whether these 21 residues are critical for sTF binding to PS. A truncated form of sTF lacking these 21 residues, TF1‒190, was generated (Fig. 5e; Supplementary information, Fig. S8h). As shown in Fig. 5f, TF1‒190 has a markedly weaker affinity to PS compared to sTF. Also, we found that the procoagulant activity of TF1‒190 is marginal compared to sTF (Fig. 5g). These results suggest that the cleavage-exposed C-terminus of sTF is critical for its procoagulant activity.

### sTF is responsible for thrombosis in the necroptosis-induced inflammation model

**Fig. 6:**
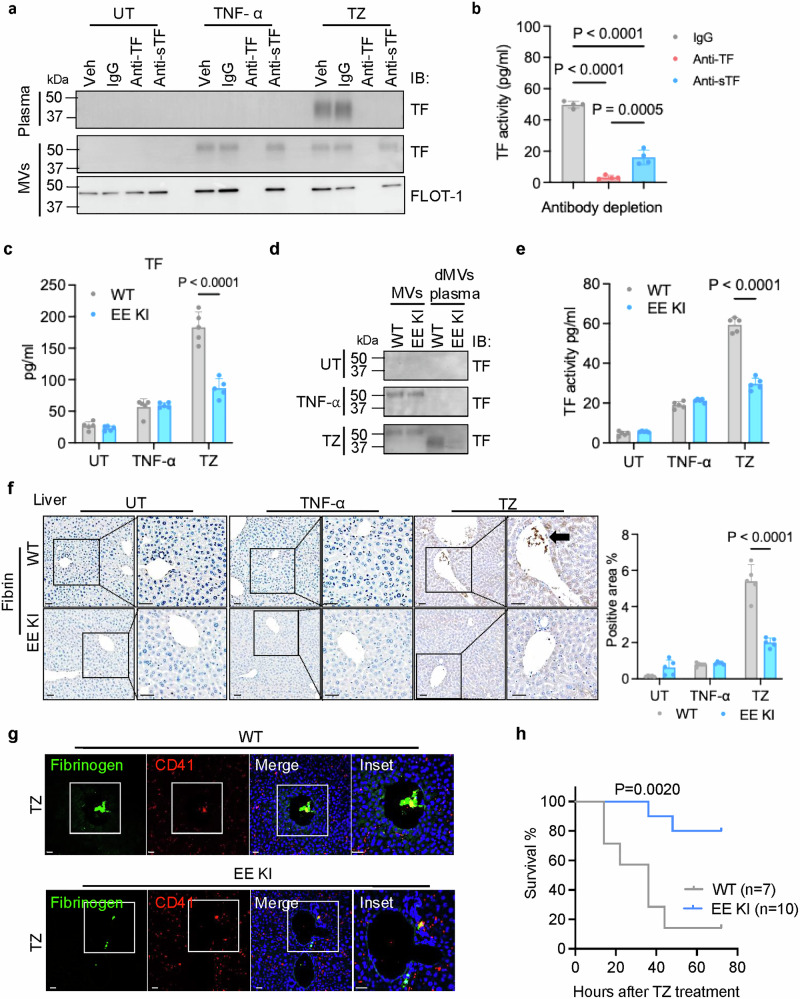
Suppressing shedding of flTF prevented thrombosis in the necroptosis-induced inflammation model. **a** TF was depleted from the plasma samples of UT, TNF-α-, or TZ-challenged WT mice using an antibody pull-down technique. The remaining plasma TF levels post either control IgG, anti-TF antibody, or anti-sTF antibody pull-down were measured by western blot. MVs were isolated from post pull-down plasma fraction and examined by western blot with indicated antibodies. **b** TF was depleted from the plasma samples of TZ-challenged WT mice by pull-down with either control IgG, anti-TF antibody, or anti-sTF antibody. The TF activity was measured in post pull-down plasma fraction by PCA assay. *n* = 4 per group. **c** The TF level in the plasma from WT or EE KI mice untreated or challenged with TNF-α alone or with TZ was measured by ELISA. *n* = 5 per group. **d** MVs were isolated from UT, TNF-α-, or TZ-challenged WT and EE KI mouse plasma by spin down at 100,000× *g* for 20 min. Isolated MVs and plasma fraction with MVs depleted were examined by western blot for TF. dMVs: MVs depleted. **e** TF activity was measured in plasma samples from WT or EE KI mice untreated or challenged with TNF-α alone or with TZ by PCA assay. *n* = 5 per group. **f** Liver sections from WT or EE KI mice untreated or challenged with TNF-α alone or with TZ were examined. Representative images of fibrin staining are shown in left panel. Fibrin IHC staining quantification was shown in right panel. Scale bars: 40 µm. **g** Liver sections from TZ-challenged WT or EE KI mice were examined. Representative images of IF staining are shown. Fibrinogen deposition was indicated by a green signal, platelets were labeled with CD41 (red), and nuclei were stained blue. Scale bars: 40 µm. **h** The survival curve of WT or EE KI mice during TZ-mediated necroptosis-induced inflammation.

A specific polyclonal antibody against sTF was developed using a short peptide contains the neo-epitope exposed by ADAM cleavage (Supplementary information, Fig. S8i). As shown in Supplementary information, Fig. S8j, the anti-sTF antibody specifically pulled down the sTF, but not the flTF, protein in immunoprecipitation experiments, whereas the anti-TF antibody pulled down both proteins. We then used this sTF-specific antibody to deplete sTF in plasma samples from TZ-treated WT mice. Both the anti-TF and our anti-sTF antibodies effectively removed sTF, the anti-TF antibody also removed the MV-associated flTF from plasma samples (Fig. 6a). While depleting with anti-TF antibody resulted in almost complete loss of TF activity, depleting only the sTF with anti-sTF antibody reduced 80% of the procoagulant activity in plasma samples from TZ-treated WT mice (Fig. 6b). This suggests that the remaining 20% activity is from the MV-associated flTF. This finding confirmed that sTF, rather than MV-associated flTF, is the primary contributor to the elevated coagulant activity observed in the plasma of TZ-treated WT mice. A similar effect of anti-sTF depletion on coagulation activity was also noted in the CM of TZ-treated J774A.1 cells (Supplementary information, Fig. S8k).

We next evaluated whether sTF is critical for thrombosis in vivo. Mice that were pre-treated with GW exhibited improved survival outcomes and faster recovery from body temperature drop in TZ-treated mice (Supplementary information, Fig. S9a, b). Administration of GW did not affect plasma LDH activity post TZ treament in these animals as compared to mice receiving vehicle control (Supplementary information, Fig. S9c). GW administration also effectively counteracted the TZ-induced hemolysis and shortening of blood clotting time (Supplementary information, Fig. S9d). The surge in plasma TF levels triggered by TZ was notably prevented by GW pretreatment (Supplementary information, Fig. S9e‒g). Consistently, GW pretreatment prevented the reduction of flTF in PBMCs and the production of sTF in plasma at 6 h post TZ challenge (Supplementary information, Fig. S9f). Administration of GW also inhibited the elevated TF activity observed in the plasma of TZ-challenged mice (Supplementary information, Fig. S9h). The TZ-triggered changes in other thrombosis indicators, fibrinogen, D-dimer and TAT were reversed by GW (Supplementary information, Fig. S9i‒k). In line with these indications, administration of GW blocked the formation of clots in both the liver (Supplementary information, Fig. S10a) and kidney (Supplementary information, Fig. S10b). Altogether, these results suggested that suppressing necroptosis-triggered TF cleavage largely blocked the production of sTF and that GW treatment inhibited thrombosis in the inflammation model.

To investigate the role of sTF in thrombosis, mice carrying knockin mutations at the ADAM cleavage site of TF (T211V212 mutated to E211E212), referred to as EE KI mice, were generated (Supplementary information, Fig. S11a). As our in vitro experiments indicated that the EE mutation of this site effectively blocked the cleavage of flTF by ADAMs without affecting TF’s coagulation activity (Fig. 4e, f), these EE KI mice allowed us to directly address the role of sTF production in thrombosis. The EE mutation in TF does not affect the populations of leukocytes in EE KI mice (Supplementary information, Fig. S11b). Also, we did not detect any difference in platelet count, size or activation in untreated WT and EE KI mice, indicating that EE mutation does not affect platelet count, size and activation (Supplementary information, Fig. S11c‒e, UT results). When WT and EE KI mice were treated with TNF-α, we observed that TNF-α induced comparable levels of TF in the plasma (Fig. 6c). However, there is a dramatic reduction of TF levels in the plasma from TZ-treated EE KI mice compared to their TZ-treated WT littermates (Fig. 6c). Western blot analysis of MVs and plasma samples from these mice showed that the level of sTF, which was present exclusively in the plasma samples of TZ-treated mice, was largely reduced in EE KI mice compared to WT mice (Fig. 6d). The sTF detected in samples of TZ-treated EE KI mice is not only present in a much lower level, but, interestingly, slightly smaller than the sTF in the plasma of TZ-treated WT mice (Fig. 6d), implying the possibility of an alternative ADAM cleavage of TF in the EE KI mice. Reanalysis of TF sequence with the MEROPS system^38^ predicts two possible, though less optimal, ADAM10/17 cleavage sites upstream of the T211/V212 cleavage site (Supplementary information, Fig. S11f). It is possible that the small amount of sTF detected in EE KI mice is generated by alternative cleavage by ADAM10/17 at one of these less optimal sites. To be sure that EE KI mutation does not alter TF expression in monocytes/macrophages, we prepared BMDMs from WT and EE KI mice. TNFα induced similar levels of elevated TF expression in both WT and EE KI BMDMs, suggesting that EE KI does not affect TNFα-induced expression of TF (Supplementary information, Fig. S11g). Also, TZ treatment caused the reduction of TF in WT, but not much in EE KI BMDMs. Therefore, these results suggested that EE mutation of TF largely blocked the cleavage of flTF and only a trivial amount of a smaller sTF was generated in TZ-treated EE KI mice.

Importantly, the dramatic increase of plasma procoagulant activity induced by TZ treatment in WT mice was significantly reduced in EE KI mice, while the minor increase in TF activity observed in plasma from TNF-α-treated EE KI mice was similar to that seen in TNF-α-treated WT mice (Fig. 6e). The procoagulant activities of isolated plasma MVs from WT and EE KI mice were similar between the two groups, regardless of treatments (Supplementary information, Fig. S11h). Additionally, the TZ-induced reductions of fibrinogen and the increases of D-dimer and TAT were largely prevented in EE KI mice (Supplementary information, Fig. S11i‒k). Thrombosis formation was also inhibited in EE KI mice following TZ treatment (Fig. 6f, g; Supplementary information, Fig. S12a‒c). Because we did not detect any significant change in platelet count at 6 h after TZ treatment, we examined platelet count, size and activation at 16 h after TZ treatment. Interestingly, we found that all of the platelet parameters are changed, and similarly in TZ-treated WT and EE KI mice (Supplementary information, Fig. S11c‒e, TZ results); however, platelet thrombus is significantly increased in 16 h TZ-treated WT, but not EE KI, mice (Supplementary information, Fig. S12d). Taken together, these findings suggest that the EE KI mutation provides substantial protection against TZ-induced thrombosis and further confirm the crucial role of sTF in thrombosis triggered by necroptosis-mediated inflammation.

Interestingly, after TZ challenge, EE KI mice exhibited dramatically improved survival and accelerated recovery from TZ-induced hypothermia compared to WT mice (Fig. 6h; Supplementary information, Fig. S12d), while plasma LDH activities were similar in EE KI and WT mice following TZ treatment (Supplementary information, Fig. S12e). These results suggest that thrombosis may play a more important role than tissue damage does in the pathogenesis of necroptosis-induced inflammation.

### Inhibiting sTF production alleviates thrombosis caused by viral infection

**Fig. 7:**
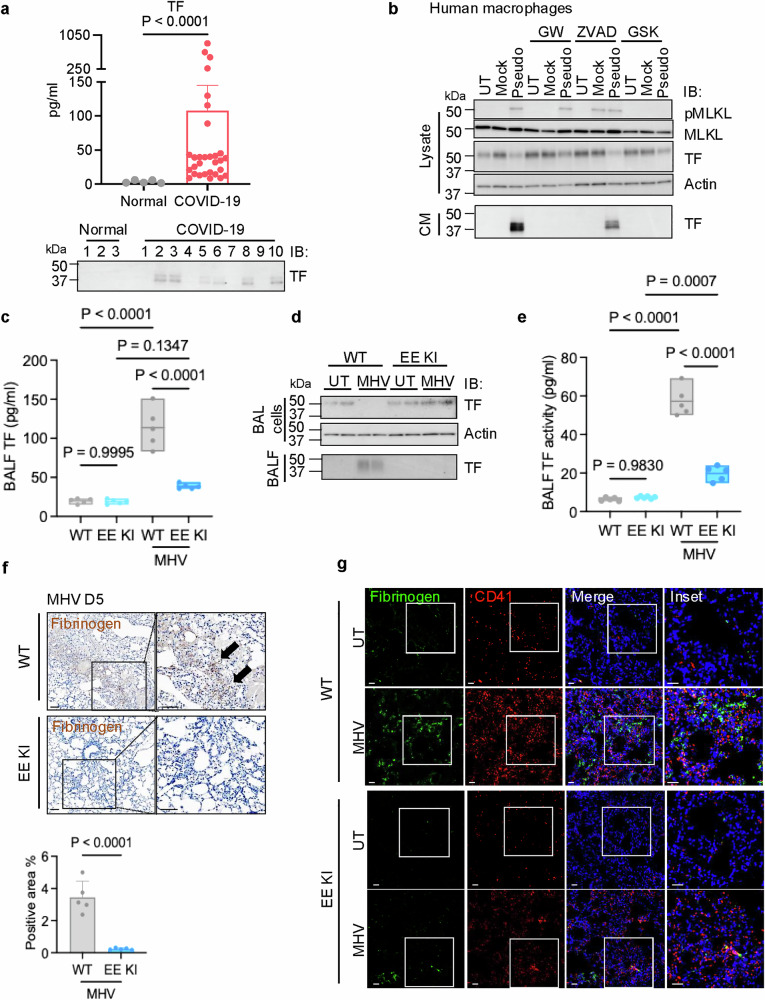
Inhibiting sTF production alleviates thrombosis caused by viral infection. **a** TF level was measured in plasma samples from either normal donors (*n* = 5) or COVID-19 patients (*n* = 30) by ELISA (upper panel). Western blot analysis of plasma from three normal donors and ten COVID-19 patients is shown in lower panel. MVs were removed from plasma samples before western blot. **b** Human primary macrophages were differentiated from elutriated monocytes and infected with mock virus or SARS-CoV-2 pseudoviral particles for 60 h. Inhibitors (GW, ZVAD, or GSK) were added 1 h prior to infection and replenished every 24 h. Lysate and CM of infected human macrophages were analyzed by western blot with the indicated antibodies. MVs were removed from CM. **c**–**g** WT and EE KI mice were intranasally inoculated with 1.5 × 10^5^ PFU MHV. Analyses were conducted on day 5 post-infection. **c** The TF level was measured in BALF from uninfected or MHV-infected WT and EE KI mice by ELISA. *n* = 5 per group. **d** BALF and BAL cells from uninfected or MHV-infected WT and EE KI mice. Samples were examined by western blot with the indicated antibodies. MVs were removed from BALF samples. **e** TF activity was examined in BALF from uninfected or MHV-infected WT and EE KI mice by PCA assay. *n* = 5 per group. **f** Representative images of uninfected or MHV-exposed lung sections from WT and EE KI mice. Representative images of fibrinogen IHC staining are shown. Fibrin IHC staining quantification was shown in lower panel. Scale bars = 40 µm. **g** Uninfected or MHV-exposed lung sections from WT and EE KI mice were examined. Representative images of IF staining are shown. Fibrinogen deposition was indicated by a green signal, platelets were labeled with CD41 (red), and nuclei were stained blue. Scale bars = 40 µm.

Based on our findings that necroptosis contributes to thrombosis through activating ADAMs to produce sTF in the mouse inflammation model, we wondered whether a similar mechanism connecting necroptosis to thrombosis is involved in viral infection. We first found that plasma TF levels of COVID-19 patients were significantly higher than that of healthy donors (Fig. 7a, top panel). Notably, elderly patients often exhibited higher levels of plasma TF ( > 50 pg/mL) (Supplementary information, Fig. S13a), aligning with the increased risk of developing thrombosis in elderly COVID-19 patients. We then evaluated the plasma TF in ten COVID-19 patients and three healthy donors by western blot and detected sTF, with a similar size to the one detected in the TZ-treated mouse plasma, in six of these patients but none of the healthy controls (Fig. 7a, bottom panel). These results implied the possibility that SARS-CoV-2 infection may lead to necroptosis of monocytes/macrophages and the subsequent production of the sTF.

To investigate this possibility, human and mouse macrophages or monocytic cell lines were infected with SARS-CoV-2 pseudoviral particles, and necroptosis and the sTF production were evaluated. As indicated by increased pMLKL levels, SARS-CoV-2 pseudovirus infection effectively induced necroptosis, which coincided with a reduction of flTF in cell lysates and a corresponding presence of sTF in the CM, while the mock virus has no such effects (Fig. 7b; Supplementary information, Fig. S13b, d, f). Treatment of either ADAM inhibitor GW or necroptosis inhibitor GSK effectively prevented the production of sTF, whereas the apoptotic inhibitor ZVAD had no effect on sTF production (Fig. 7b; Supplementary information, Fig. S13c, e, f). These results suggested that SARS-CoV-2 infection resulted in necroptosis of monocytes/ macrophages and the production of sTF.

To assess these findings in an in vivo viral infection model, we employed intranasal infection with MHV-A59 (MHV) in mice (Supplementary information, Fig. S14a).^42,43^ The level of TF in bronchoalveolar lavage fluid (BALF) increased progressively, reaching a peak on the 5th day post-MHV infection (Supplementary information, Fig. S14b). Hence, for evaluating the role of necroptosis and sTF in MHV model, all sample collections were carried out on Day 5 post infection unless specified otherwise. IF imaging showed that MHV infection induced necroptosis in mononuclear BAL cells, as evidenced by pMLKL staining (Supplementary information, Fig. S14c). Examination of lung tissue from MHV-infected mice revealed that pMLKL-positive cells resembled F4/80-positive macrophages, indicating that necroptosis primarily occurs in macrophages (Supplementary information, Fig. S14d). To confirm that necroptosis-mediated generation of sTF is from macrophages, we used *TF*^*fl*/*fl*^ and *LysM*-*Cre*;*TF*^*fl*/*fl*^ mice in the MHV infection model. When we examined the BALF from these mice at 5-days post-infection, we found that the production of sTF is not detected in *LysM*-*Cre*;*TF*^*fl*/*fl*^ mice (Supplementary information, Fig. S14e).

MLKL KO or administration of GW did not significantly alter MHV titer, inflammatory responses, or BAL cell composition on the 5th day post-MHV infection (Supplementary information, Fig. S14f‒h). However, MHV infection in WT mice resulted in nearly undetectable levels of flTF in BAL cells, with a significant increase of sTF in the BALF, which was blocked by GW administration or MLKL KO (Supplementary information, Fig. S15a, b). Importantly, MHV infection stimulated TF activity in the BALF of WT mice, which was abolished by GW pretreatment or MLKL KO (Supplementary information, Fig. S15c). Extensive clot formation was observed in the lungs of MHV-infected WT mice and was markedly reduced in GW-pretreated WT mice or MLKL KO mice (Supplementary information, Fig. S15d, e).

Similarly, TF levels in BALF were significantly reduced in MHV-challenged EE KI mice (Fig. 7c). The decrease of flTF in BAL cells was largely mitigated in EE KI mice compared to WT mice following MHV infection (Fig. 7d). While sTF levels were elevated in the BALF of WT mice after 5 days of MHV infection, they were not detected in MHV-infected EE KI mice (Fig. 7d). TF activity in the BALF increased by 5-fold in WT mice post-MHV infection, but this increase was markedly reduced in EE KI mice (Fig. 7e). Extensive clot formation observed in the lungs of MHV-infected WT mice was significantly diminished in EE KI mice (Fig. 7f, g; Supplementary information, Fig. S15f). These results strongly indicate that sTF production is crucial for viral infection-induced thrombosis.

## Discussion

The hemostatic system serves the dual purposes of regulating blood flow in normal circumstances and initiating blood clot formation to safeguard the host from vascular damage. Thrombosis occurs when there is abnormal blood clot formation under pathological conditions like necroptosis-induced inflammation and viral infections. TF is a critical regulator for both hemostasis and thrombosis. As TF is a membrane protein, it is believed that the membrane flTF is responsible for initiating blood coagulation under physiological and pathological conditions. To date, there is no evidence that a functional sTF can mediate blood coagulation under either hemostatic or thrombotic conditions. Our findings identified the first functional sTF, which is generated by necroptosis-mediated shedding of the membrane flTF. This sTF is distinct from the EctoTF and is functional in initiating blood coagulation and importantly, is responsible for thrombosis under the conditions of necroptosis-induced inflammation and viral infection. In contrast to the current understanding of TF function in thrombosis, we demonstrated that the membrane flTF has a limited role in thrombosis under these pathological conditions. Our study not only revealed a critical role of sTF in thrombosis, but also provided a diagnostic marker of thrombosis and potential therapies for treating necroptosis-induced inflammation or viral infection-caused thrombosis, while not affecting hemostasis, by targeting the sTF, necroptosis or ADAMs.

TF is known to be predominantly expressed in subendothelial tissues under physiological conditions and its expression could also be potently induced in monocytes and macrophages under pathological conditions by endotoxin or inflammatory cytokines.^44^ Since no soluble TF, such as the asTF, has been found to participate in blood coagulation, it is widely believed that the membrane flTF is the major player in hemostasis and thrombosis. The increased plasma levels of TF observed under pathological conditions such as viral infection was thought to be from elevated TF-containing MVs.^45,46^ Notably, previous studies found that monocytes/macrophages play a critical role in thrombosis and are thought to be the source of TF-containing MVs.^12,47^ In our study, we identified the first functional sTF that is shed from the membrane flTF on necroptotic monocytes/macrophages by the cell surface proteases ADAMs (Figs. 2–6). Mapping the ADAM10/17 cleavage site in flTF allowed us: (1) to demonstrate that the sTF is dramatically different from the EctoTF and is fully functional in mediating coagulation although has slightly lower activity than the flTF (Fig. 5b–d; Supplementary information, Fig. S8e, f). In order for TF to efficiently convert FVII to FVIIa, TF needs to bind to phospholipid platform. Unlike flTF, the ectoTF does not have the transmembrane domain and does not bind to phospholipid well. However, the cleavage of TF generates a unique C-terminal end that is highly hydrophobic and has a RK positive charge cluster and allows sTF to bind phospholipid effectively as flTF does. While this region is present in ectoTF, the C-terminal disulfide bond (Cys190‒Cys213) in ectoTF likely prevents this region to be exposed to phospholipid and therefore, ectoTF is a poor phospholipid binding peptide. Also, the results of PT assays and Supplementary information, Fig. S8g suggest that the phospholipid (e.g., PS) in plasma is sufficient for sTF to be fully functional and the blood cells may not play a major role in direct modulation of sTF coagulation activity; (2) to generate sTF-specific antibody and to demonstrate that sTF, not the MV-associated flTF, is responsible for the elevated procoagulant activities in plasma of TZ-treated WT mice and CM of TZ-treated J774A.1 cells (Fig. 6a, b; Supplementary information, Fig. S8h‒j); (3) to generate EE KI mice with mutations of this ADAM10/17 cleavage site in TF and to confirm the critical role of sTF in thrombosis in both inflammation and viral infection models (Figs. 6c–h and 7c–g). The complete blockage of TF cleavage by ADAMs in vitro, while a trivial amount of sTF produced in TZ-treated EE KI mice, is likely because ADAM10/17 activity and expression vary between MEF cells (used in the in vitro assay) and primary cells in the EE KI mice. Interestingly, we barely detected any sTF in MHV-infected EE KI mice (Fig. 7d), suggesting that the type and the potency of different necroptosis inducers may be relevant for the alternative cleavage of TF by ADAM10/17 in EE KI mice.

Intriguingly, while the induction of flTF on monocytes/macrophages is similar in mice treated with TNF-α or TZ (MLKL deletion in Fig. 3a, or GW in Supplementary information, Fig. S9f), a notable difference was observed in the development of thrombosis (Fig. 1f, g; Supplementary information, Figs. S3a, b and S10a, b). While TNF-α-induced flTF on the cell membrane may remain predominantly inactive, the cleavage of flTF to sTF could potentially act as an activation mechanism for TF in the context of necroptosis-related thrombosis. Also, as MVs isolated from plasma of TNF-α- or TZ-treated mice presented similar coagulation activities (Fig. 2g, h), it is likely that flTF on MVs was not activated by TZ treatment either. The data from EE KI mice further supported that the cleavage of flTF is the key regulation to elevate the procoagulant activity of TF in both necroptosis-induced inflammation and viral infection models (Figs. 6c–e and 7c–e).

Fibrin deposition and platelet thrombus formation are two key components of thrombosis. In our inflammation model (TZ), we observed significant fibrin deposition after 6 h of TZ treatment, but no obvious platelet aggregation until later stage (16 h) of TZ treatment. Interestingly, while platelet aggregation, detected in TZ-treated WT mice, was dramatically reduced in TZ-treated EE KI mice (Supplementary information, Fig. S12d), the decrease of platelet count was similar in both TZ-treated WT and EE KI mice (Supplementary information, Fig. S11c). As inflammation can cause thrombocytopenia, both thrombosis and inflammation may contribute to the changes of platelet count in TZ model. Also, because the direct binding of sTF to platelets is not detectable, it is unlikely that sTF directly recruits platelets or promote platelet production.

Inflammation and viral infection contribute to the pathogenesis of necroptosis and thrombosis.^27,48^ Given our findings that thrombosis is dependent on necroptosis and that inhibiting thrombosis significantly improves prognosis and reduces inflammation and viral infection severity in mouse models (Figs. 6f, g and 7f, g), we propose that necroptosis contributes to the pathology of these conditions by promoting thrombosis through the production of sTF. Specifically, necroptosis and thrombosis have been reported in COVID-19 patients.^31,33,49–51^ In fact, we detected elevated levels of sTF in the blood samples of human COVID-19 patients (Fig. 7a). Consistent with our animal studies, we found that when human monocytes/macrophages undergo necroptosis following TSZ or SARS-CoV-2 pseudovirus infection, the membrane flTF is shed to produce fully functional sTF (Fig. 7b; Supplementary information, Figs. S8a‒c and S13b‒f). As there is relatively high (60%) homology between mouse and human flTF proteins and the mouse and human sTF have similar sizes, we believe that the mouse and human sTF are produced similarly by ADAMs during necroptosis. While our work strongly suggests that sTF is primarily produced from necroptotic monocyte/macrophage under inflammation and viral infection conditions, it is worth noting that as ADAM proteases could be activated by other membrane disturbance in addition to necroptosis, it is possible that sTF might also be produced from other types of cells including endothelial cells in non-necroptotic conditions. These possibilities need to be evaluated in other pathological conditions. A report of sTF with similar size being detected in the blood of cancer patients supports this possibility.^52^

Thrombosis plays a pivotal role in numerous pathological conditions, making prompt diagnosis and effective interventions essential to mitigate disease progression.^31,53^ The current thrombosis marker is D-dimer, a fibrin degradation byproduct that appears following the breakdown of blood clots by fibrinolysis.^54^ However, D-dimer is not a specific marker for thrombosis. Several physiological and pathological conditions (i.e., aging and pregnancy), not necessarily characterized by thrombus formation, have been associated with increased D-dimer.^55,56^ In contrast, this newly identified sTF could serve as a diagnostic marker for the pre- or initial coagulation phase of thrombosis, given its role as an upstream initiator of coagulation cascade during necroptosis-induced inflammation and viral infection.

The current treatment for thrombosis, such as administration of heparin, affects both hemostasis and thrombosis by inactivating both thrombin and FXa which can result in the side effect of excessive bleeding.^57^ In contrast, targeting necroptosis or ADAMs will only block thrombosis by inhibiting sTF production and will not impede hemostasis. Taken together, our study not only revealed a critical role of this sTF in thrombosis, but also provides a diagnostic marker of thrombosis and potential therapies for treating necroptosis-induced inflammation or viral infection-caused thrombosis by targeting the sTF, necroptosis or ADAMs.

## Materials and methods

### Mice

8- to 12-week-old male FVB/J mice were purchased from Jackson Laboratory and MLKL KO FVB/J mice were obtained from our in-house breeding.^58^ All mice were bred and housed in specific pathogen-free conditions with free access to food and water. All animal experiments were performed under protocols approved by National Cancer Institute Animal Care and Use Committee and followed NIH guidelines.

### Generation of mTF E211E212 knockin mice

The point mutation mouse model at exon 5 (211 T to E, 212 V to E) of the mouse TF gene was generated via CRISPR-Cas9 technology. Briefly, Cas9 mRNA and guide RNA were produced by in vitro transcription, an oligonucleotide donor DNA was synthesized and the mixture of Cas9 mRNA, gRNA and donor DNA was microinjected into fertilized eggs (FVB/J). The gRNA used was: GTGCACACTGTACTGCTTCC. The donor oligos were: TTGTACAAGCTATGATTTTCTCCAGGAAAACTAACCAAAATAGCCCAGGAAGCAGTGAGGAATGCACCGAGCAATGGAAGAGTTTCCTGGGAGGTGAGTGGCCATGGCTGTCAGTGCCC. The site of target for mouse TF E211E212 knockin (EE KI) genotypes are shown in Supplementary information, Fig. S11a. All homozygous TF EE KI mice used for analysis in this project are males at 8‒12-weeks old.

### Generation of *LysM*-*Cre*;*TF*^*fl*/*fl*^ conditional knockout mice

TF^*fl*/*fl*^ and *LysM*-*Cre*^*Cre*/*Cre*^ mice were obtained from the Jackson Laboratory and cross-bred to generate *LysM*^*WT*^/^*Cre*^;*TF*^*fl*/Δ^ mice which were then bred to obtain TF littermate control (*LysM*-*Cre*^−/−^;*TF*^*fl*/*fl*^) or TF KO (*LysM*-*Cre*^+^;*TF*^*fl*/*fl*^) mice for deletion in myeloid cells (macrophages and neutrophils). To genotype these mice: *TF*^*fl*/*fl*^ mice were identified by genotyping using the primers: Forward: 5’-GTCCATTCCCAGCATCCA-3’ and Reverse: 5’-CACTTGGCACCCCACTCC-3’. Mice that are TF homozygous for the flox were selected and genotyped for the expression of LysM-Cre using the primers; Forward: 5′-CTTGGGCTGCCAGAATTCTC-3′; Reverse: 5’-CCCAGAAATGCCAGATTACG-3′. All animals were housed under specific pathogen-free conditions at the National Institutes of Health in an American Association for the Accreditation of Laboratory Animal Care-approved facility.

### Necroptosis-induced inflammation mouse model

Mice were administered murine tumor necrosis factor-alpha (TNF-α; R&D, aa 80‒235, 6 μg/200 μL/mouse) by intravenous (i.v.) injection, either as a standalone treatment or in conjunction with z-VAD-FMK (ZVAD; R&D, FMK001). For combined treatment, ZVAD was injected intraperitoneally (i.p.) 15 min before (250 μg/200 μL PBS/mouse) and again 1 h after (100 μg/200 μL PBS/mouse) TNF-α injection.^59^ All solutions were prepared using endotoxin-free phosphate buffered saline (PBS). Body temperature and survival were monitored every 3 h. Mice were euthanized with CO_2_ inhalation. Blood samples were collected from the tail vein in citrate (135 mM, 1:9 volume with blood) and corn trypsin inhibitor (final concentration of 20 mg/mL in blood) preloaded tubes at the indicated times. Isolated plasma was also used in ELISA and procoagulant activity assays.

### Necroptosis blockage in vivo

Nec-1 (full name, 5-((1H-indol-3-yl) methyl)-3-methyl-2-thioxoimidazolidin-4-one) was purchased from Selleckchem (S8037). Nec-1 (125 μg, i.v.) was given 17 min before TNF-α injection.^60^

### ADAM activity inhibition in vivo

For inhibition of ADAM activity in TZ model, FVB/J male mice were i.p. administered with vehicle control or 100 μg/kg GW 280264X (R&D, 7030) 24 h and 48 h before TNF-α or TNF-α/ZVAD (TZ) treatment.^25,58^

### Monocyte/macrophage depletion in mice

Mice were administered 0.2 mL clodronate liposome or control liposomes (Clophosome®) via i.p. injection 48 h and 24 h prior to the TZ treatment.^61^ The depletion efficiency was confirmed by flow cytometry analysis of mouse spleen cells.

### Mouse cytokine array profiling

Cytokine production in murine TZ models were measured with Proteome Profiler Mouse XL Cytokine Array (R&D, ARY028). 200 μL plasma samples were used in this multiplex antibody array. Blot analysis and quantitation was conducted following standard procedures.

### Histology analysis

At the indicated time points, mice received transcardiac perfusion with 10 unit/mL heparin/PBS. Once perfusion is completed, kidney, liver, or lung tissues were collected immediately. Samples were fixed with Z-fix (Anatech) for 24 h before further histology analysis. The fixed tissues were embedded in paraffin and cut into 5-μm serial sections. For H&E analysis, tissue sections were stained with hematoxylin and eosin according to standard procedures. For immunohistochemistry analysis, fixed tissue sections were deparaffinized and subjected to antigen retrieval. Citrate retrieval buffer (pH 6.0, Dako, S2369) was employed for the staining of fibrin (Sigma-Aldrich, MABS2155, 1:100), fibrinogen (Dako, A0080, 1:200) and pMLKL (Abcam, ab196436, 1:1000),^62^ whereas Tris-EDTA buffer (pH 9.0, Abcam, ab93684) was utilized for antigen retrieval of F4/80 (eBioscience, 14-4801-82, 1:500) staining. Following incubation with both primary and secondary antibodies, the sections were exposed to a streptavidin-horseradish peroxidase complex. DAB was applied for color development, and a counterstain of hematoxylin was administered prior to microscopic evaluation. Quantitative analysis was conducted using ImageJ software.

### IF staining of thrombi in tissue

Tissue cryosections were examined for thrombi formation via IF for the presence of platelets (CD41, BD Pharmingen, 561850, 1:100) and fibrin(ogen) (Dako, A0080, 1:200). Nuclei were stained using 4,6-diamidino-2-phenylindole (DAPI, Invitrogen, P36935). Images were acquired using a laser scanning confocal microscope (Zeiss LSM 780). Images were processed by Zen (Zeiss, blue edition, version 3.9) software.

### Blood clotting time in capillary tubes

After anesthesia with isoflurane inhalation, blood was collected into plain capillary tubes (~100 μL/tube) through retro-orbital bleeding.^63^ Filled capillary tubes were kept at 37 °C and broken every 30 s. When a clot starts forming with fiber present, that is the endpoint and clotting time.^64^

### Human monocyte isolation and macrophage differentiation

Human monocytes were isolated using counter-flow elutriation, as previously described.^65,66^ Post-isolation, cells were pelleted and subjected to a 10-min treatment with ACK Lysing Buffer (Lonza, Walkersville, MD, USA) to remove erythrocytes. Following this, cells were washed and resuspended in RPMI-1640 medium (Gibco), supplemented with 10% (v/v) fetal bovine serum (FBS), 2 mM glutamine, 100 U/mL penicillin, and 100 μg/mL streptomycin.

For the differentiation process, the culture medium was then supplemented with 50 ng/mL human macrophage colony-stimulating factor (M-CSF, R&D, 216-MC), with additional supplementation every three days, for a total duration of six days.

### PBMC isolation

Peripheral blood from mice was obtained as described above. PBMCs were separated by density gradient centrifugation over Lympholyte Mammal (Cedarlane Laboratories Ltd, CL5115) at 800× *g* for 30–40 min at room temperature, followed by triplicate washes, per manufacturer’s protocol.^67^

### BMDMs

Mouse bone marrow cells were obtained from tibia and femur of 8–10-week-old FVB/J male mice and subsequently cultured in complete DMEM supplemented with mouse M-CSF (R&D, 416-ML) at a concentration of 15 ng/mL. Mouse M-CSF was supplementated every three days. After 7 days, mouse BMDMs were divided and plated in 12-well tissue plate for 24 h before treatment.

### In vitro treatment of macrophages and monocytic cell lines

Culture media were removed, and the plates were rinsed twice with PBS. Serum-free media were then applied prior to treatment. J774A.1 cells were pretreated with 20 µM ZVAD for 30 min followed by 30 ng/mL mouse TNF-α for 6 h to induce necroptosis. In mouse BMDMs, U937 cells, and human macrophages, necroptosis was triggered by 20 µM ZVAD and 10 nM Smac mimetic for 30 min followed by 30 ng/mL mouse or human TNF-α (TSZ) for 6 h.

Pharmacological modulators, including 3 µM GSK’872 (GSK, Selleckchem, S8465), 40 µM Necrostatin-1 (Nec-1, Selleckchem, S8037), or 10 µM GW, were administered 1 h prior to TNF-α treatment. Post-treatment, cells were collected and instantly lysed using 1× SDS buffer for western blot analysis.

For the collection of CM, cellular debris was first eliminated from the culture supernatant through centrifugation. The resulting supernatant were concentrated 1000-fold utilizing a 10 K spin column (Amicon, UFC5010) and reserved for subsequent experiments.

### TF activity assay

Adapted from previously established methods, the activation of FX by human FVIIa and murine TF was assessed with continuous PCA assay.^68,69^ All the materials and plate reader should be pre-warmed to 37 °C before assay. A 5 µM concentration of Phosphatidylcholine: Phosphatidylserine (PC:PS) vesicles (Haematex, X9113) was mixed with the CM sample and incubated for 30 min at room temperature before analysis. Plasma samples (1:10 diluted in HBSA), isolated MVs (resuspended in HBSA), or PC:PS-pretreated CM samples were co-incubated with human FVIIa (final concentration of 2.4 nM) and human FX (Prolytix, HCX-0050, final concentration of 73.2 nM) in an HBSA buffer enriched with 5 mM CaCl_2_ at 37 °C for 2 h. To terminate FXa generation, 25 µL of HBSA containing 25 mM EDTA was added to achieve a final EDTA concentration of 5 mM. The mixture was allowed to incubate for 5 min at room temperature.

For the subsequent stage, transfer 80 µL of the mixture from the initial stage to a fresh well on a black plate, duplicating this setup. Introduce 20 µL of FXa-specific fluorogenic substrate (Prolytix, SN-7) to reach a final concentration of 0.5 mM in an HBSA buffer containing 5 mM CaCl_2_. Mix thoroughly and measure fluorescence emission at 470 nm (with excitation at 352 nm) in 1-min increments over a span of 60 min at 37 °C. The fluorescence tracing curves were recorded and maximal rate of substrate hydrolysis (V_max) was calculated by identifying the steepest incline in the linear segment of the fluorescence progress curve.^70^ The standard curve was generated with lipidated human TF standards (Abcam, ab108906), and by regression analysis of the 4-parameter curve with Graphpad Prism 10. TF activity of samples was interpolated using the sample readings from the linear part of the standard curve.

### TF depletion

Plasma samples (100 µL) were diluted with 900 µL of PBST and pre-cleaned by pull-down with protein G beads for 1 h on a rocker at 4 °C to remove any existing antibodies. TF was then depleted from the pre-cleaned plasma or CM samples by incubating with anti-mTF antibody (ab189483, 1 µg/mL) or anti-sTF antibody (1 µg/mL) for 2 h on a rocker at 4 °C, followed by a 1-h pull-down with protein G beads. Rabbit IgG was used as a control.

### MV isolation

To isolate MVs from plasma, 100 μL of plasma was diluted in 900 μL of HBSA (137 mM NaCl, 5.38 mM KCl, 5.55 mM glucose, 10 mM HEPES, 0.1% bovine serum albumin, pH 7.5). Cellular debris was first eliminated from the diluted plasma or CM samples through centrifugation. MVs were then pelleted from plasma at 100,000× *g* centrifugation for 20 min at 4 °C, or at 20,000× *g* centrifugation for 20 min at 4 °C from CM. The pellet was washed once with 1 mL of HBSA and re-suspended in 100 μL HBSA for procoagulant activity or directly lysed with 1× SDS buffer for western blot analysis.

### mTF construct generation and in vitro cleavage

pLV-Puro-CMV>mouse TF[NM_010171.3]/EGFP/Myc was ordered from Vector builder. Mouse asTF DNA was ordered from IDT DNA, and subcloned into pLV-Puro-CMV vector.

Full-length mouse TF (flTF)/GFP/HA, E211E212 mutated mouse TF (EEmutTF-GFP-HA, flTF/HA, Ectodomain mouse TF (aa 1‒223) (EctoTF-HA)), EEmutTF/HA, cleaved soluble mouse TF (aa 1‒211) (sTF-HA), alternatively spliced mouse TF (asTF-HA), and C-terminal tail truncated sTF (TF1‒190-HA) were generated using Q5 Site-Directed Mutagenesis Kit (NEB, E0554S). All the constructs were validated by Sanger sequencing (Diagrams shown in Figs. 4d and 5a, e).

Mouse flTF/GFP/HA and EEmutTF/GFP/HA constructs were transfected into MEF cells with electroporation (Nucleofector®, Lonza). 8 h post transfection, 3 μM GSK, 40 μM Nec-1, or 10 μM GW were added 1 h before TNF-α treatment. Cells were then treated with TSZ for 16 h.

### TF protein purification and relipidation

Mouse flTF/ HA, EctoTF/HA, TF sTF/HA, EEmutTF/HA, asTF/HA, or TF1‒190/HA plasmid was transfected into HEK293 cells. 24 h post transfection, cell lysate was collected with M2 lysis buffer. HA-tagged TF protein was pulled down by anti-HA magnetic beads (Thermo Fisher Scientific, 88836) and washed with M2 lysis buffer for five times. Binding protein was eluted with 100 μL of 2 mg/mL HA peptide (Thermo Fisher Scientific, 26184) for three times. Combined elution product was relipidated through octylglucoside (OG) dialysis. Briefly, 2.6 µmol of PC:PS vesicles, at an 80:20 molar ratio, were resuspended in 400 µL of a freshly prepared OG/HBS solution. The eluted recombinant mTF was then added. After thorough mixing, the mixture was incubated at room temperature for 30 min and then dialyzed against three changes of HBS.^71^ The finalized product was analyzed in western blot and PCA assay.

For the PCA assay, the same amount of relipidated mouse flTF-HA, EctoTF-HA, TF sTF-HA, EEmutTF-HA, asTF-HA, or TF1‒190-HA protein (10 pg/assay) was examined as stated above in “TF activity assay” section, unless otherwise specified.

### Lipid binding assay

To assess the lipid binding affinity of mouse TF proteins, a protein-lipid overlay assay was performed using Membrane Lipid Strips (Echelon Biosciences, P-6003) according to the manufacturer’s protocol. Membranes were first blocked in 3% (w/v) fatty acid-free BSA (Sigma-Aldrich) in PBST (0.1% (v/v) Tween 20) for 1 h at room temperature, protected from light. After blocking, the membranes were incubated overnight at 4 °C with TF proteins (flTF-HA, EctoTF-HA, sTF-HA, asTF-HA, or TF1‒190/HA) purified from HEK293 cells, at a concentration of 1.0 μg/mL in PBST with gentle agitation. Following incubation, membranes were washed four times with PBST over a 30-min period to remove unbound protein. The membranes were then incubated for 1 h with mouse anti-HA polyclonal antibody (Invitrogen, #26183) at 1 μg/mL, followed by a 1-h incubation with anti-mouse-HRP conjugate (1:10,000 dilution). Lipid-bound TF proteins were detected using enhanced chemiluminescence (ECL).

### PT

Recombinant mouse TF proteins, including flTF-HA, EctoTF-HA, sTF-HA, or asTF-HA were purified from HEK293 cells and relipidated. For measurements of the procoagulant function of relipidated mouse TF protein, coagulation was initiated by mixing 50 μL of plasma and 50 μL TF solution containing 25 mmol/L CaCl_2_ and the clotting time was determined by the Semi-automatic Coagulation Analyzer (LABOMED, SCO-2004).

### Anti-sTF antibody generation and validation

The polyclonal antibody against mouse sTF was developed with GenScript. The antigen peptide (mTF aa 192‒211) was illustrated in Supplementary information, Fig. S8i. The immunization was performed in two New Zealand White rabbits. From the immunized animals, the sera were isolated, and polyclonal antibody was purified by affinity chromatography. The binding affinity of the antibody to sTF and flTF was assessed using a pull-down assay, following the same procedures described in the “TF depletion” method, using Rabbit IgG control, anti-TF antibody (ab189483), and the anti-sTF antibody. The plasma samples depleted of TF or sTF were then evaluated for PCA activity to determine the effectiveness of the antibody.

### COVID-19 patients’ plasma collection and evaluation

Single donor human SARS-CoV-2 positive plasma from Innovative Research (IPLASCOV2PVP100UL) and Raybiotech (CoV-PosA-P-100) were collected through an IRB-approved protocol with ethics approval and full donor consent to participate, and COVID-19 positive status has been confirmed via SARS-CoV-2 IgG antibody assay and/or PCR positive test results. Plasma from healthy donors were obtained from NIH blood bank. Samples were processed using EDTA as the anticoagulant and inactivated with 4.0% Triton X-100. TF level was also measured in inactivated plasma by ELISA.

### SARS-CoV-2 pseudovirus infection

Cells were infected with Ad5CMV-SARS-CoV2-Spike (U of Iowa-7643) pseudoviral particle at M.O.I 50. 3 μM GSK, 4 μM Nec-1, or 10 μM GW were added 1 h before infection and replenished every 24 h. Following the 48-h incubation period, cells were washed twice with PBS and subsequently cultured in serum-free medium for an additional 12 h. Ad5CMV-mCherry (VVC-U of Iowa-649) was used as a mock control and to indicate the infection efficiency. 60 h post infection, both infected cell lysate and CM was collected for further analysis.

### Mouse MHV-A59 model

WT, TF EE KI, and MLKL KO mice, aged between 8 and 12 weeks, were used in the study. All experimental procedures involving animals were approved by the National Cancer Institute Animal Care and Use Committee (IACUC) and conducted in accordance with NIH guidelines. Mouse Hepatitis Virus A59 (MHV-A59) viral stock was obtained from BEI Resources (NR43000). The viral stock was stored at ‒80 °C until needed.

For ADAM activity inhibition, WT mice were administered with 100 μg/kg GW (i.p.) immediately prior to the MHV-A59 intranasal inoculation, 2 days post-inoculation, and again 4 days post-inoculation.

Mice were anesthetized using isoflurane inhalation to minimize stress and discomfort. Once anesthetized, each mouse was intranasally inoculated with 15 × 10^3^ PFU of MHV-A59 virus (in 20 μL) using a pipette, ensuring even distribution of the inoculum between both nostrils.^42^ Following the inoculation, animals were closely monitored for any signs of distress, weight loss, or other disease symptoms. At indicated time points post-inoculation, mice were humanely euthanized for sample collection. Lung and BAL samples were collected for further analysis.

### MHV N gene PCR

MHV-A59 N gene expression in the lung tissue was analyzed by RT-PCR. Briefly, total RNA was extracted from 100 mg lung tissue with RNeasy Mini kit (Qiagen, 74004). 500 ng total RNA was used for reverse transcription with PrimeScript™ RT reagent Kit (Takara, RR037B). PCR reactions were then performed using the primers shown in Table 1. PCR products (123 bp) were resolved by electrophoresis in 2% agarose gels and visualized with a Bio-Rad imaging system.

**Table 1:** RT-PCR primers

	Forward 5’-3'	Reverse 5’-3'
MHV N gene	CAGATCCTTGATGATGGCGTAGT	AGAGTGTCCTATCCCGACTTTCTC
L32	AAGCGAAACTGGCGGAAAC	TAACCGATGTTGGGCATCAG

### BALF collection

BALF was collected following published protocol.^72^ In brief, 0.8 mL of PBS were injected into lungs through a 22 G catheter in the trachea. Then the fluid was aspirated out slowly. This process was repeated for a second round. The recovered lavage fluid was centrifuged at 800× *g* for 10 min at 4 °C. Only the supernatant from the first round of lavage was saved as BALF.

### BAL cells analysis

The collected BALF was centrifuged at 800× *g* for 10 min at 4 °C to pellet the cells. The pellet from two rounds of lavage fluid was combined and subjected to red blood cell lysis. A cell sample containing 4.0 × 10^5^ cells was diluted with 200 μL PBS and cytospun onto a slide. Slides were air dried at room temperature overnight. Cells were fixed with cold methanol for 10 min. After blocking and being permeabilized with 10% normal goat serum (0.05% v/v Triton X-100), cells were incubated with anti-pMLKL antibody (Cell Signaling Technology, clone D6E3G; 1:100), followed by secondary antibodies. Afterwards, cells were stained with DAPI to visualize nuclei. Imaging was carried out using Zeiss LSM 780. May-Grünwald Giemsa staining in BAL cells was conducted according to standard procedures.^73^

For composition analysis in BAL cells post MHV infection, cells were stained with a panel of fluorescently-conjugated antibodies specific to cell surface markers for different cell types (e.g., macrophages, neutrophils, T cells, etc.), as per the manufacturer’s instructions.^74^ After a 30-min incubation at 4 °C, cells were washed twice with PBS. Stained cells were analyzed using a BD^TM^ LSR Fortessa SORPII. At least 10,000 events were recorded for each sample. Data were analyzed using Flowjo software (Tables 2, 3, and 4). The percentage and absolute numbers of each cell type were calculated. For the strategy of analysis refer to Supplementary information, Fig. S16.

**Table 2:** Fluorescently conjugated antibody panel

Marker	Color/Format	Antibody volume
CD45	APC	0.5 μL
CD11b	Brilliant Violet 605	0.5 μL
CD3	Alexa Fluor 488	0.5 μL
CD19	APC-Cy7	0.5 μL
Ly-6G (GR-1)	PE	0.5 μL
CD11c	PE-Cy7	0.1 μL
MHC II	Pacific Blue	0.5 μL
Fixable Live/Dead Aqua	Fixable Live/Dead Aqua	0.5 μL

**Table 3:** Primary antibodies used in western blot

Antibody	Manufacturer	Dilution
Anti-mouse TF antibody	Abcam, ab189483	1:500
Anti-mouse MLKL (phospho S345) antibody	Abcam, ab196436	1:1000
Anti-MLKL antibody	Abcam, ab184718	1:1000
Anti-human TF antibody	R&D, AF2339	1:500
Anti-human pMLKL (phospho S358) antibody	Abcam, ab187091	1:1000
HA antibody	Invitrogen, 26183	1:2000
Mouse FVII antibody	R&D, AF3305	1:2000
Flotillin-1 Antibody	CST, 3253	1:1000
β-Actin antibody	Sigma, A2066	1:2000
sTF antibody #16	Genscript Custom	1:1000

**Table 4:** Fluorescently conjugated antibody panel for leukocyte analysis

Marker	Color/Format	Antibody volume
CD45	APC	1:200
CD11b	PE	1:200
CD3	Alexa Fluor 488	1:200
CD4	PE-Cy7	1:200
CD8	PerCP-Cy5.5	1:200
CD19	APC-Cy7	1:200
Ly-6G	Brilliant Violet 711	1:200
SiglecF	Brilliant Violet 421	1:200
Ly-6C	Brilliant Violet 605	1:200
Fixable Viability Stain 440UV	Fixable Viability Stain 440UV	1:1000

### IF imaging of J774A.1 cells

J774A.1 cells were seeded on ibidi dishes overnight before treatment. After TNF-α or TZ treatment, cells were fixed in 4% paraformaldehyde for 15 min then permeabilized by 0.25% Triton X-100/PBS. After blocking with 10% normal goat serum, cells were incubated with anti-TF antibody (Abcam, ab189483, 1:100) and anti-F4/80 antibody (eBioscience, #14-4801-82, 1:200), followed by secondary antibodies. Afterwards, cells were stained with DAPI to visualize nuclei. Imaging was carried out using Zeiss LSM 780.

### Cytometric bead array (CBA) mouse inflammation analysis

The BD™ CBA Mouse Inflammation Kit (552364) was used to quantitatively measure Interleukin-6 (IL-6), Interleukin-10 (IL-10), Monocyte Chemoattractant Protein-1 (MCP-1), Interferon-γ (IFN-γ), TNF-α, and Interleukin-12p70 (IL-12p70) protein levels in mouse BALF samples. 5 μL of BALF was used in each assay. CBA analysis was performed on BD™ LSR Fortessa and analyzed with Flowjo. Data analysis and quantitation was conducted following BD™ manual.

### ELISA assays

Mouse plasma concentrations of TAT (Abcam, ab137994), Fibrinogen (Abcam, ab108844), and D-dimer (Novus, NBP3-08100) were measured using commercial ELISA kits. The TF level was measured in plasma, CM, and BALF with Mouse Coagulation Factor III/TF DuoSet ELISA kit (R&D, DY3178-05), linear range of the TF activity assay in a quantitative manner is 0–750 pg/mL, as determined by the standard curve of recombinant flTF. Human Coagulation Factor III/Tissue Factor Immunoassay (R&D, DCF300) was used to examine the human plasma samples. The concentration was calculated according to the standard curve.

### LDH activity assay

LDH activity assay was done in mouse plasma samples with LDH Activity Assay kit (Sigma-Aldrich, MAK066) to evaluate the presence of tissue damage. The concentration was calculated according to the NADH standard curve.

### Protein de-glycosylation

1‒20 µg of glycoprotein was denatured by heating in Glycoprotein Denaturing Buffer (NEB, B0701S) at 100 °C for 10 min. Glycoprotein was chill denatured and 1 µL PNGase F (NEB, P0704) was added. Reaction was incubated at 37 °C for 1 h. De-glycosylation reaction was inhibited with 1× SDS loading dye and ready for western blot analysis.

### Platelet count measurement

Whole blood(~100 μL) was collected via retro-orbital bleeding into Eppendorf tubes pre-loaded with 2 μL of 0.5 M EDTA (pH 8.0), and platelet counts were measured using an Element HT5^TM^ veterinary hematology analyzer (Heska) according to the manufacturer’s instructions.

### Western blotting

To detect TF, 100 µL of citrated plasma (pre-spin, or post-spin) or 400 µL of BALF was processed using High Select™ Depletion Spin Columns (Thermo Scientific, A36369) to decrease the concentration of high-abundance proteins before western blot. The flow-through from three mice in the same experimental group was pooled and concentrated to 100 µL using a 10 K spin column (Amicon, UFC5010), and then 20 µL was used for western blot. All the isolated MVs from three mice were combined and directly lysed with 1× SDS buffer for western blot.

For conditioned media (CM), 10 mL from 2 × 10^7^ cells were concentrated to 100 µL with a 10 K spin column and 20 µL was used in western blot. All the isolated MVs were combined and used for western blot.

The samples were subjected to electrophoresis and separated by SDS-PAGE, and then transferred to polyvinylidene fluoride membranes (EMD Millipore). Due to the low protein abundance of TF, the blots were visualized with SuperSignal™ West Femto Maximum Sensitivity Substrate (Thermo Scientific, 34095) on Bio-Rad imaging system.

### Leukocyte immunophenotyping analysis

Cellular components were pelleted from 100 µL citrated whole blood, and red blood cells were lysed using ACK Lysing Buffer. The leukocytes were then washed to remove debris and resuspended in PBS. After FC block for 20 min at 4 °C, the cells were stained with a panel of fluorescently conjugated antibodies specific to various cell surface markers (e.g., macrophages, neutrophils, T cells) for 30 min at 4 °C. The stained cells were analyzed using a BD™ LSR Fortessa SORPII flow cytometer, recording at least 50,000 events per sample. Data analysis was performed using FlowJo software to determine the percentage of each cell type. See Supplementary information, Fig. S16a for the gating strategy.

### Flowcytometry analysis of platelets

For the percentage of platelet in whole blood, 20 μL of the citrated whole blood were diluted in 200 μL staining buffer (1% BSA in PBS) and 20 µL of the diluted whole blood was used for staining. Each sample was stained with CD41-PE (BD Pharmingen, 561850, 1:200 in 100 µL staining buffer). See Supplementary information, Fig. S16b for the gating strategy.

For platelet activation analysis, platelet-rich plasma (PRP) was prepared from citrated blood by centrifugation at 300× *g* for 20 min. 50 µL PRP was diluted with Tyrode’s solution and stained with CD41-PE (BD Pharmingen, 561850, 1:200 in 100 µL staining buffer) and CD62P-Alexa Fluor 647 (BD Pharmingen, 563674, 1:200 in 100 µL staining buffer). The collected samples were analyzed using a BD™ LSR Fortessa SORPII flow cytometer. Data analysis was performed using FlowJo software. See Supplementary information, Fig. S16c for the gating strategy.

## Supplementary information

Fig. S1

Fig. S2

Fig. S3

Fig. S4

Fig. S5

Fig. S6

Fig. S7

Fig. S8

Fig. S9

Fig. S10

Fig. S11

Fig. S12

Fig. S13

Fig. S14

Fig. S15

Fig. S16

## Data Availability

All biological materials are available upon reasonable requests under material transfer agreements (MTA) with National Cancer Institute, National Institutes of Health.
