# Supplementary material for: Soluble tissue factor generated by necroptosis-triggered shedding is responsible for thrombosis
Source: Cell Res. 2025 Sep 12;35(11):840–58. doi: 10.1038/s41422-025-01167-8 (PMC12589612; doi:10.1038/s41422-025-01167-8)
Supplement: Supplementary file 1 — Fig. S1 [file 41422_2025_1167_MOESM1_ESM.pdf]

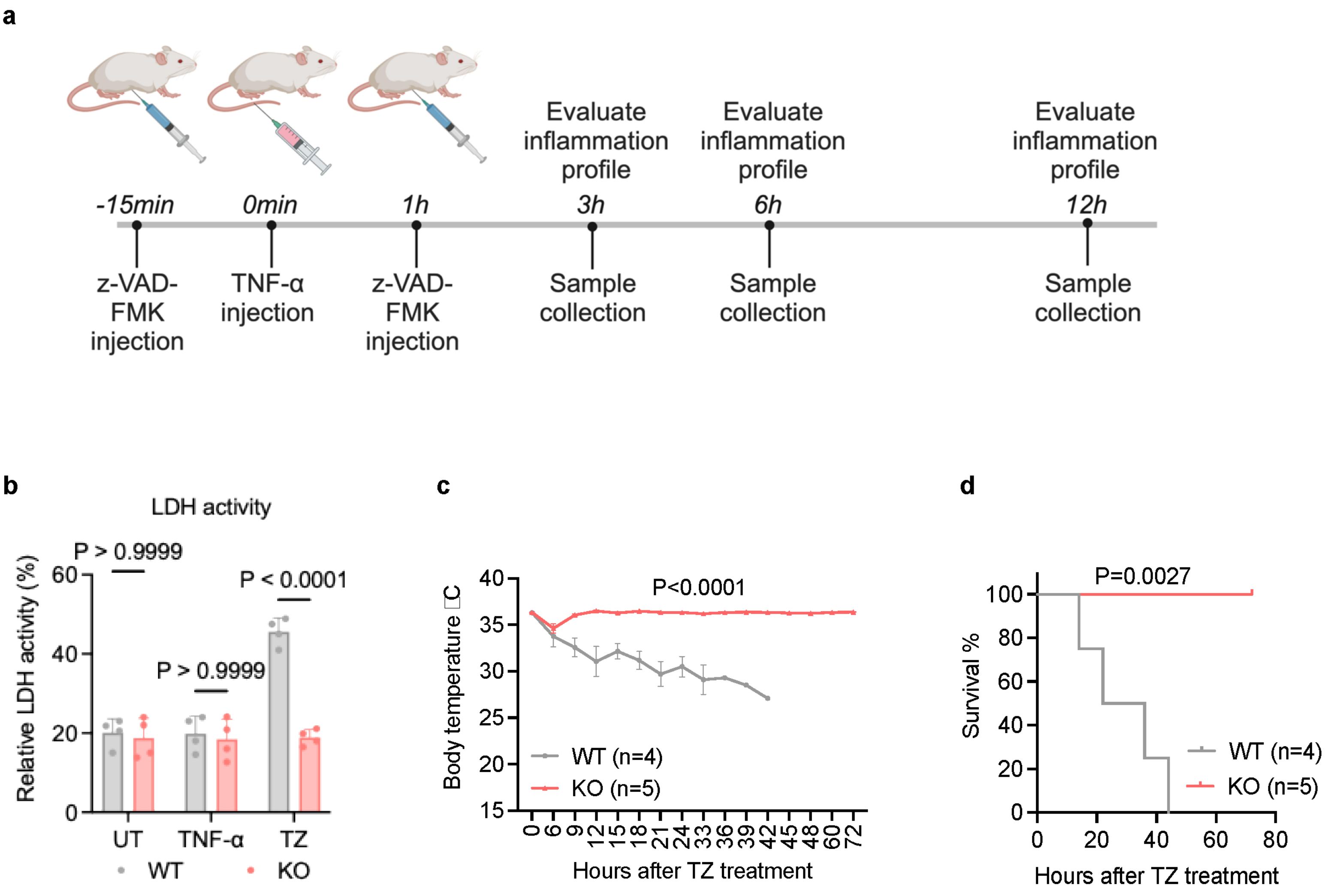

Supplementary information, Fig. S1 Necroptosis-induced inflammation model

a Diagram of necroptosis-induced inflammation model. FVB/J WT and MLKL KO mice received ZVAD (i.p.) 15min before and one hour after the TNF-α (i.v.) injection. Samples were collected at 3h, 6h, and 12h post TNF-α administration.

b LDH activity was measured in plasma from untreated, TNF-α, or TZ-challenged WT and MLKL KO mice at 6h post treatment. n=4 per group.

c Body temperature change in WT and MLKL KO mice after TZ challenge.

d Survival curve of WT and MLKL KO mice post TZ challenge.
