# Supplementary material for: Soluble tissue factor generated by necroptosis-triggered shedding is responsible for thrombosis
Source: Cell Res. 2025 Sep 12;35(11):840–58. doi: 10.1038/s41422-025-01167-8 (PMC12589612; doi:10.1038/s41422-025-01167-8)
Supplement: Supplementary file 2 — Fig. S2 [file 41422_2025_1167_MOESM2_ESM.pdf]

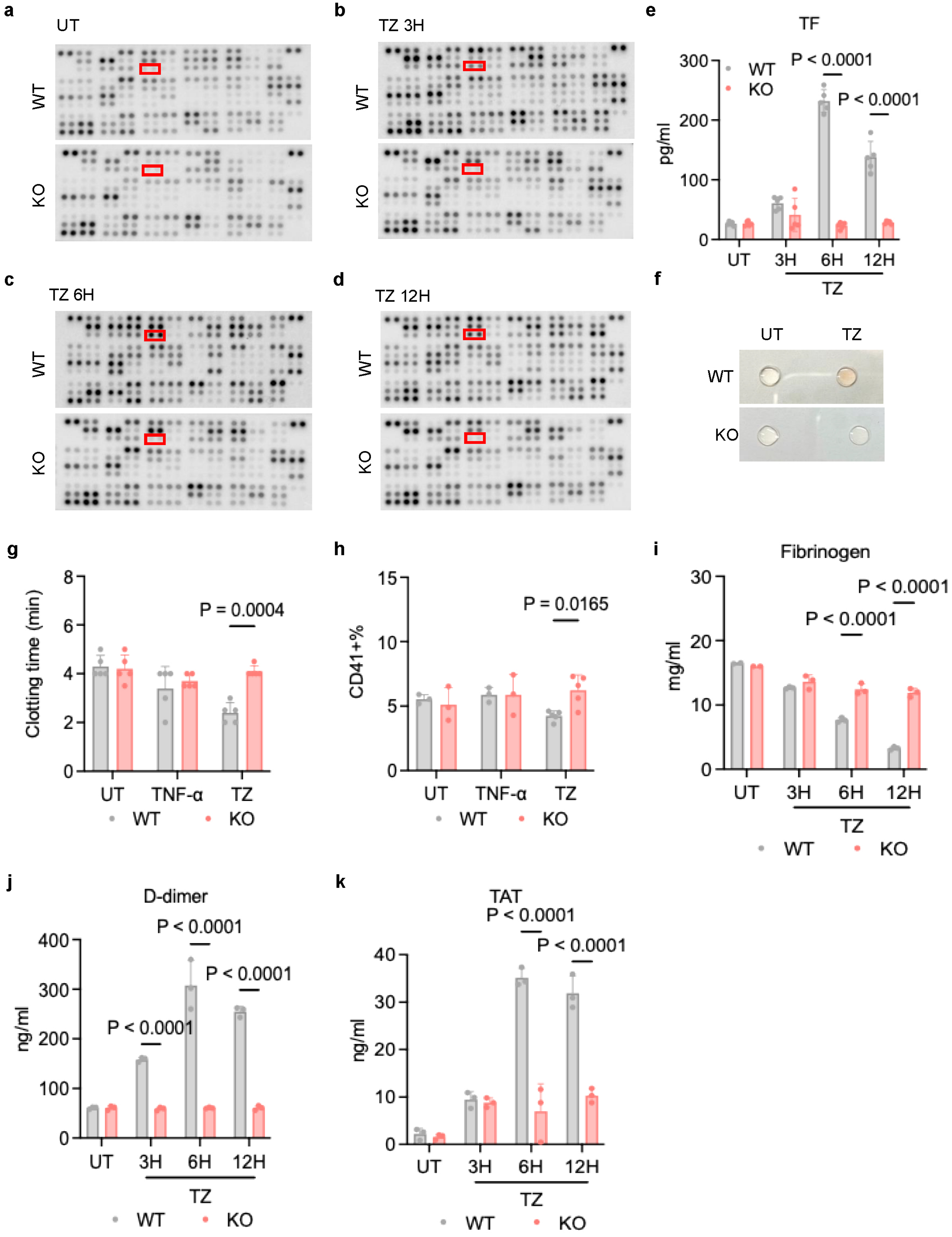

a
UT
WT
KO
b
TZ 3H
WT
KO
e
TF
WT
KO
P < 0.0001
P < 0.0001
pg/ml
300
200
100
0
UT
3H
6H
12H
TZ
c
TZ 6H
WT
KO
d
TZ 12H
WT
KO
f
UT
TZ
WT
KO
g
Clotting time (min)
8
6
4
2
0
P = 0.0004
UT
TNF-α
TZ
WT
KO
h
CD41+%
15
10
5
0
P = 0.0165
UT
TNF-α
TZ
WT
KO
i
Fibrinogen
mg/ml
30
20
10
0
P < 0.0001
P < 0.0001
UT
3H
6H
12H
TZ
WT
KO
j
D-dimer
ng/ml
400
300
200
100
0
P < 0.0001
P < 0.0001
P < 0.0001
UT
3H
6H
12H
TZ
WT
KO
k
TAT
ng/ml
40
30
20
10
0
P < 0.0001
P < 0.0001
UT
3H
6H
12H
TZ
WT
KO

Supplementary information, Fig. S2 Hypercoagulation in necroptosis-induced inflammation model

a-d Plasma samples from WT and MLKL KO mice without treatment or at 3h, 6h, and 12h post TZ challenge were collected. Mouse inflammation cytokine array was carried out with these plasma samples. Representative arrays are shown here.

e Plasma levels of TF from WT and MLKL KO mice without treatment or at 3h, 6h, and 12h post TZ challenge by ELISA assay. n=5 per group.

f Image of hemolysis as assessed by color change in plasma samples from WT and MLKL KO mice without treatment or at 6h post TZ challenge. Representative images are shown here.

g Plasma samples were collected from WT and MLKL KO mice without treatment or at 6h post TNF-α or TZ challenge. Clotting time of these plasma samples were measured in capillary tubes. n=5 per group.

h Whole blood samples were collected from WT and MLKL KO mice without treatment or at 6h post TNF-α or TZ challenge in citrate tubes. Whole blood samples were analyzed for platelets by flowcytometry with CD41 staining immediately after collection. n=3 in UT or TNF-α challenged groups, n=5 in TZ challenged group.

i-k Plasma levels of Fibrinogen, D-dimer, and TAT from WT and MLKL KO mice without treatment or at 3h, 6h, and 12h post TZ challenge by ELISA assay. n=3 per group.
