# Supplementary material for: Soluble tissue factor generated by necroptosis-triggered shedding is responsible for thrombosis
Source: Cell Res. 2025 Sep 12;35(11):840–58. doi: 10.1038/s41422-025-01167-8 (PMC12589612; doi:10.1038/s41422-025-01167-8)
Supplement: Supplementary file 3 — Fig. S3 [file 41422_2025_1167_MOESM3_ESM.pdf]

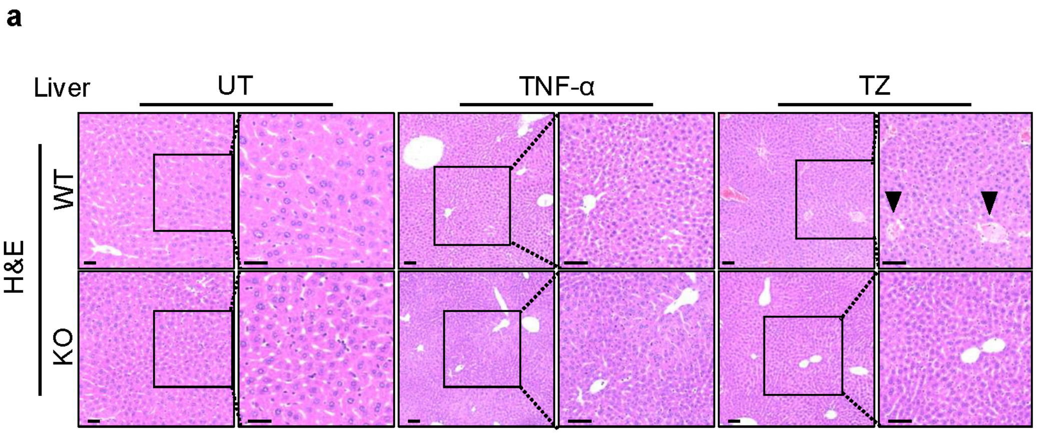

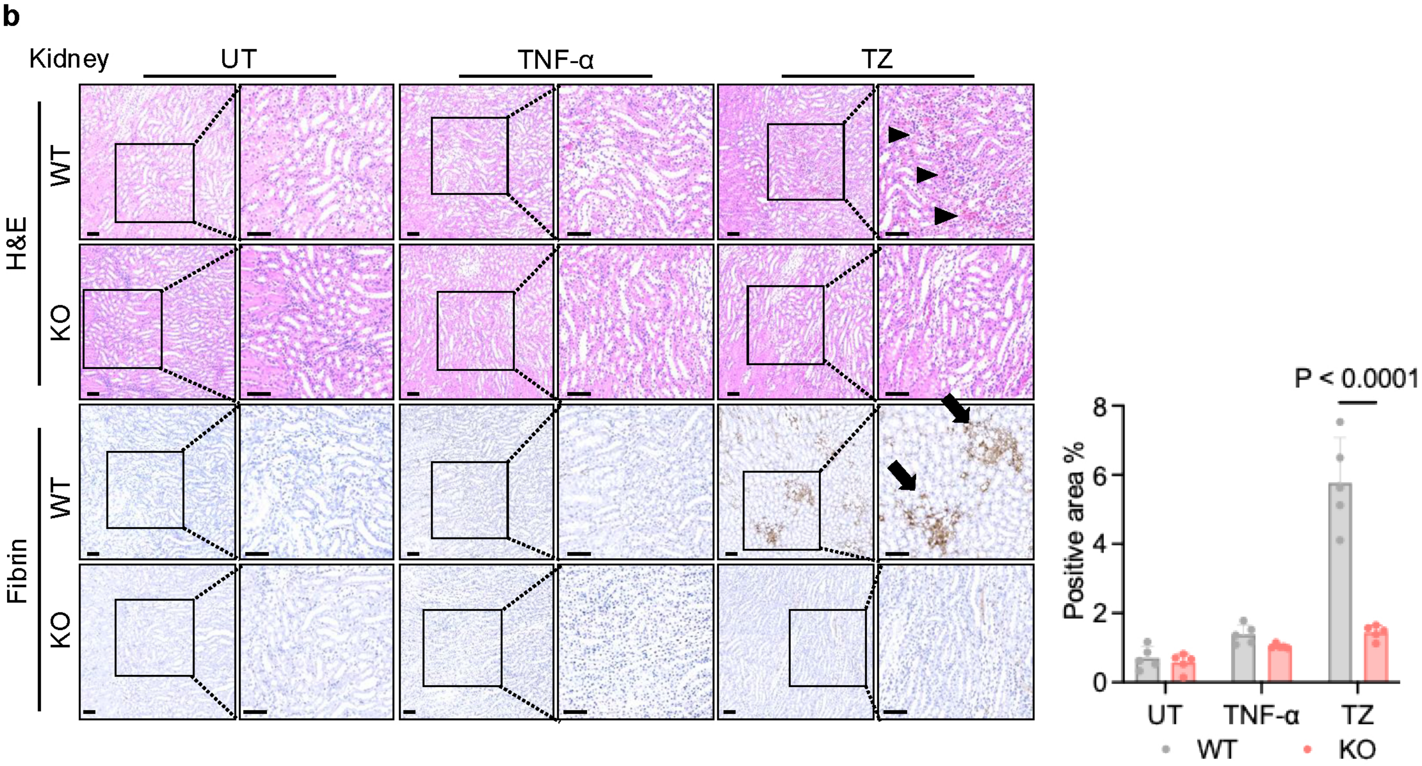

Supplementary information, Fig S3. Thrombosis in necroptosis-induced inflammation model

a Representative images of H&E staining of liver sections from untreated, TNF-α, or TZ challenged WT and MLKL KO mice at 6h post treatment. Scale bar=40μm. Arrowhead: thrombus.

b Representative images of H&E staining (upper left panel) and fibrin IHC (lower left panel) of kidney sections from untreated, TNF-α, or TZ challenged WT and MLKL KO mice at 6h post treatment. Right panel, fibrin IHC staining quantification is shown here. Scale bar=40μm. Arrowhead: thrombus. Arrow: fibrin signal.
