# Supplementary material for: Soluble tissue factor generated by necroptosis-triggered shedding is responsible for thrombosis
Source: Cell Res. 2025 Sep 12;35(11):840–58. doi: 10.1038/s41422-025-01167-8 (PMC12589612; doi:10.1038/s41422-025-01167-8)
Supplement: Supplementary file 4 — Fig. S4 [file 41422_2025_1167_MOESM4_ESM.pdf]

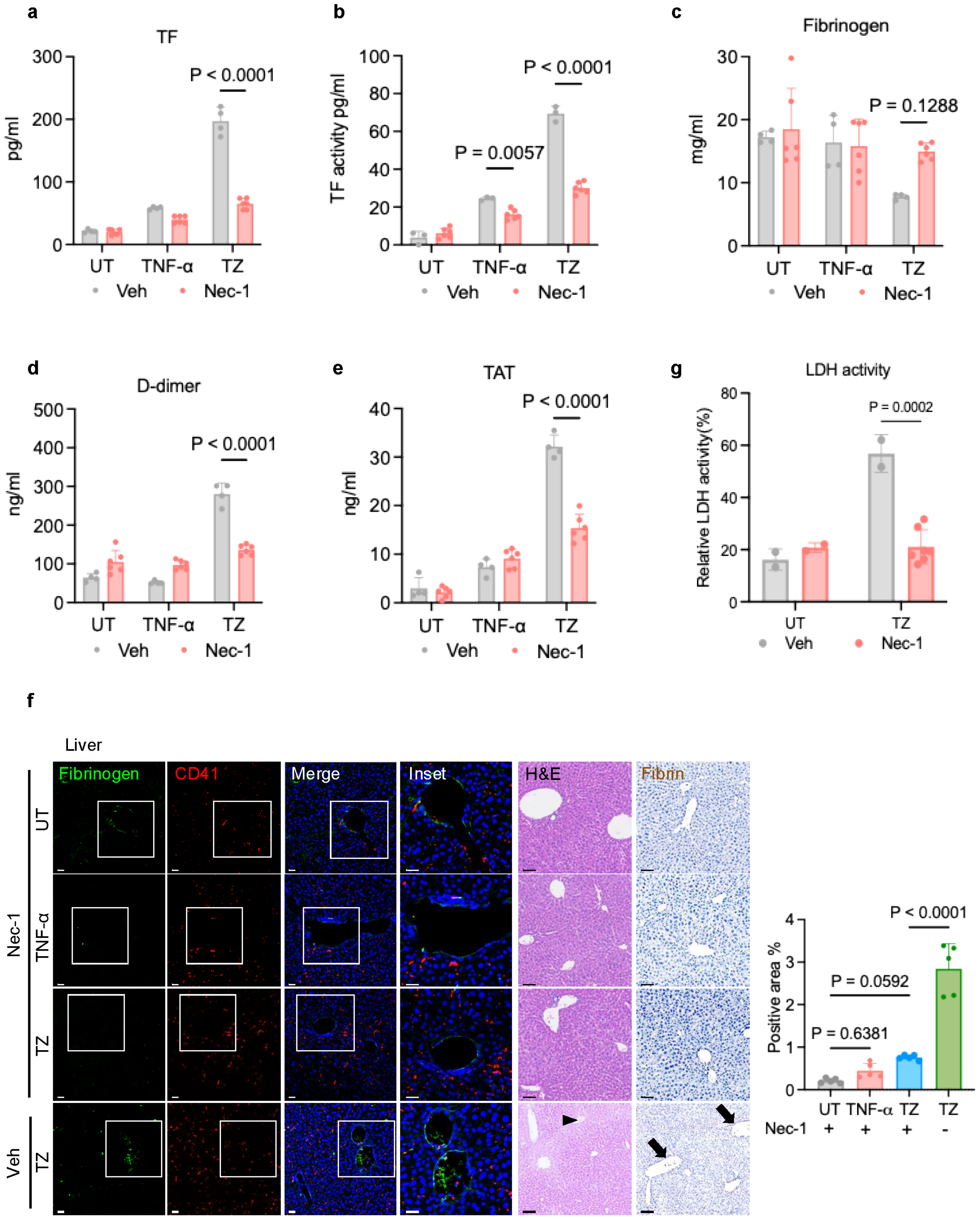
a
TF
pg/ml
300
200
100
0
P < 0.0001
UT
TNF-α
TZ
Veh
Nec-1
b
TF activity pg/ml
100
80
60
40
20
0
P = 0.0057
P < 0.0001
UT
TNF-α
TZ
Veh
Nec-1
c
Fibrinogen
mg/ml
30
20
10
0
P = 0.1288
UT
TNF-α
TZ
Veh
Nec-1
d
D-dimer
ng/ml
500
400
300
200
100
0
P < 0.0001
UT
TNF-α
TZ
Veh
Nec-1
e
TAT
ng/ml
40
30
20
10
0
P < 0.0001
UT
TNF-α
TZ
Veh
Nec-1
g
LDH activity
Relative LDH activity(%)
80
60
40
20
0
P = 0.0002
UT
TZ
Veh
Nec-1
f
Liver
Fibrinogen
CD41
Merge
Inset
H&E
Fibrin
Nec-1
UT
TNF-α
TZ
Veh
TZ
Positive area %
4
3
2
1
0
P < 0.0001
P = 0.0592
P = 0.6381
UT
TNF-α
TZ
TZ
Nec-1
+
+
+
-

Supplementary information, Fig. S4 Inhibiting necroptosis blocked TZ induced thrombosis

FVB/J WT mice received either vehicle (Veh) or necrostatin-1 (Nec-1, i.v.) at 17min before TNF-α injection. ZVAD (i.p.) was given at 15min before and one hour after the TNF-α (i.v.) injection. Samples were collected at 6h post TNF-α administration.

a Plasma levels of TF were measured using an ELISA assay in mice pre-treated with either Veh or Nec-1 and then challenged with TNF-α or TZ. n=4 in Veh groups, n=6 in Nec-1 groups.

b Plasma TF activity was measured using an PCA assay in mice pre-treated with either Veh or Nec-1 and then challenged with TNF-α or TZ. n=3 in Veh groups; n=6 in Nec-1 groups.

c-e Plasma levels of Fibrinogen, D-dimer, or TAT was measured using an ELISA assay in mice pre-treated with either Veh or Nec-1 and then challenged with TNF-α or TZ. n=4 in Veh groups; n=6 in Nec-1 groups.

f Representative images of IF staining, H&E staining, and fibrin IHC of liver sections from mice pre-treated with either Veh or Nec-1 and then challenged with TNF-α or TZ. In IF images (four left columns), fibrinogen deposition was indicated by a green signal, platelets were labeled with CD41 (red), and nuclei were stained blue. In IHC panel (far right column), fibrin deposition was in brown signal. Arrowhead:thrombus. Arrow: fibrin signal. Scale bar=40μm. Quantification of fibrin IHC staining images.

g LDH activity was measured in mice pre-treated with either Veh or Nec-1 and then challenged with or without TZ. Untreated with vehicle or Nec-1 and TZ + Veh treated groups n=2; TZ+Nec-1 group n=7.
