# Supplementary material for: Soluble tissue factor generated by necroptosis-triggered shedding is responsible for thrombosis
Source: Cell Res. 2025 Sep 12;35(11):840–58. doi: 10.1038/s41422-025-01167-8 (PMC12589612; doi:10.1038/s41422-025-01167-8)
Supplement: Supplementary file 5 — Fig. S5 [file 41422_2025_1167_MOESM5_ESM.pdf]

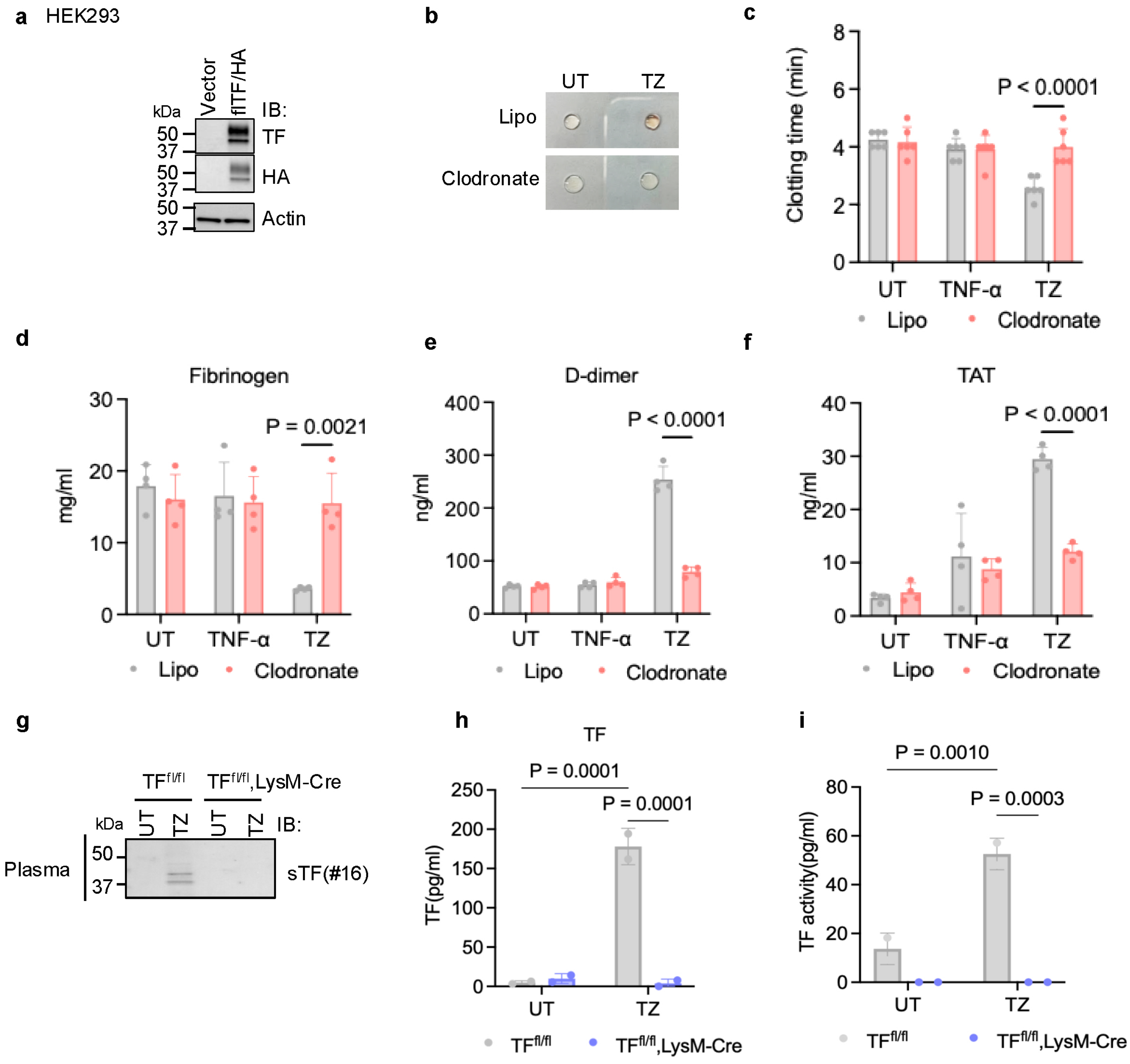

Supplementary information, Fig. S5 Monocytes/macrophages are responsible for the production of the sTF

a Empty vector or flTF/HA plasmid was transfected into HEK293 cells for 24h. Lysate was collected and examined with WB with indicated antibodies.

b-f WT mice received 0.2ml control liposomes (Lipo) or clodronate liposome via intraperitoneal injection 48h and 24h prior to the TZ treatment. All the assays were done at 6h post TZ treatment.

(b) Image of hemolysis as assessed by color change in plasma samples from Lipo or clodronate pre-administered mice without treatment or post TZ challenge. Representative images are shown here.

(c) Clotting time of plasma samples from Lipo or clodronate pre-administered mice either untreated or treated with TNF-α or TZ were measured in capillary tubes. n=6 per group.

d-f ELISA assay for (**d**)Fibrinogen, (**e**)D-dimer, and (**f**)TAT levels in plasma. Plasma samples from UT, TNF-α alone, or TZ-challenged Lipo or clodronate pre-administered mice were examined. n=4 per group.

g-i Levels of plasma sTF from $TF^{fl/fl}$ and $TF^{fl/fl}$,LysM-Cre mice with or without TZ treatment for 6 h analyzed by **(g)**WB blotting or (**h**)tissue factor ELISA. (**i**)The coagulation activity of these samples was measured by PCA assay.
