# Supplementary material for: Soluble tissue factor generated by necroptosis-triggered shedding is responsible for thrombosis
Source: Cell Res. 2025 Sep 12;35(11):840–58. doi: 10.1038/s41422-025-01167-8 (PMC12589612; doi:10.1038/s41422-025-01167-8)
Supplement: Supplementary file 6 — Fig. S6 [file 41422_2025_1167_MOESM6_ESM.pdf]

a

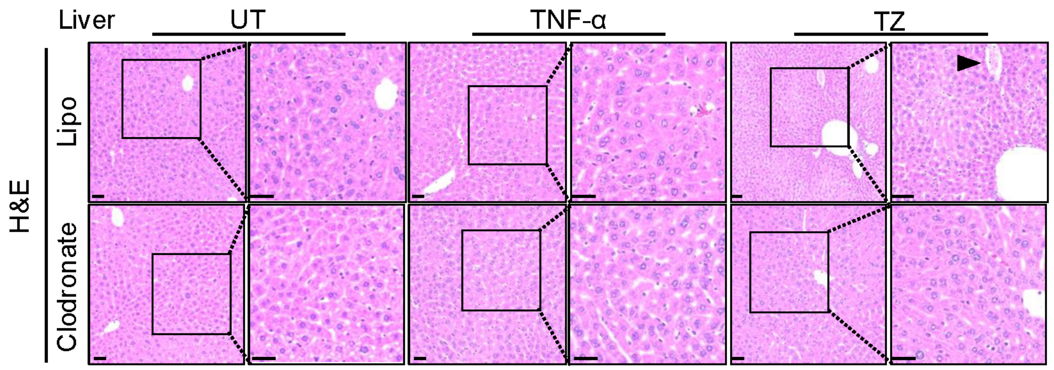

b

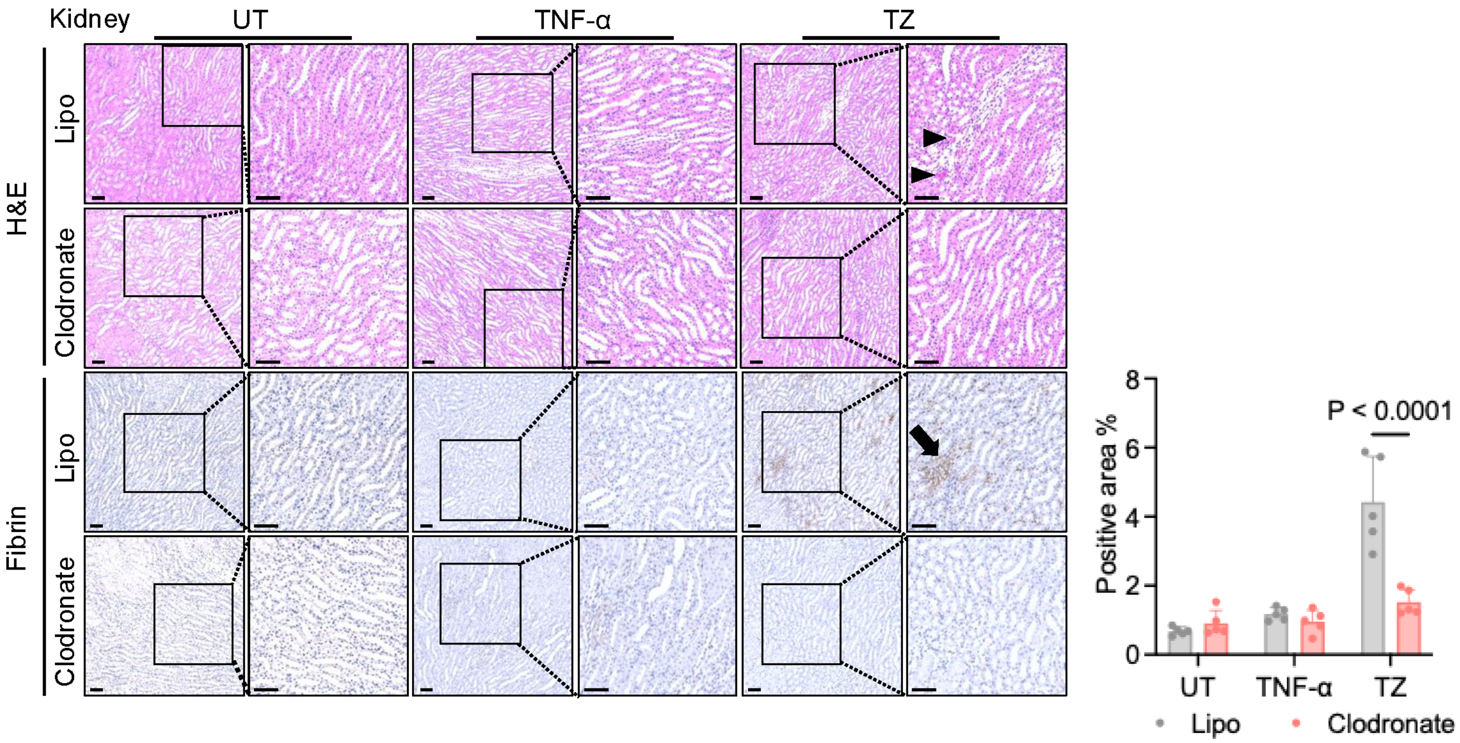

Supplementary information, Fig S6. Monocytes/macrophages are responsible for thrombosis in TZ model

a Representative images of H&E staining of liver sections from UT, TNF-α alone, or TZ-challenged Lipo or clodronate pre-administered mice. Scale bar=40μm. Arrowhead: thrombus.

b Representative images of H&E staining (upper left panel) and fibrin IHC (lower left panel) of kidney sections UT, TNF-α alone, or TZ-challenged Lipo or clodronate pre-administered mice. Right panel, fibrin IHC staining quantification is shown here. Scale bar=40μm. Arrowhead: thrombus. Arrow: fibrin signal.
