# Supplementary material for: Soluble tissue factor generated by necroptosis-triggered shedding is responsible for thrombosis
Source: Cell Res. 2025 Sep 12;35(11):840–58. doi: 10.1038/s41422-025-01167-8 (PMC12589612; doi:10.1038/s41422-025-01167-8)
Supplement: Supplementary file 7 — Fig. S7 [file 41422_2025_1167_MOESM7_ESM.pdf]

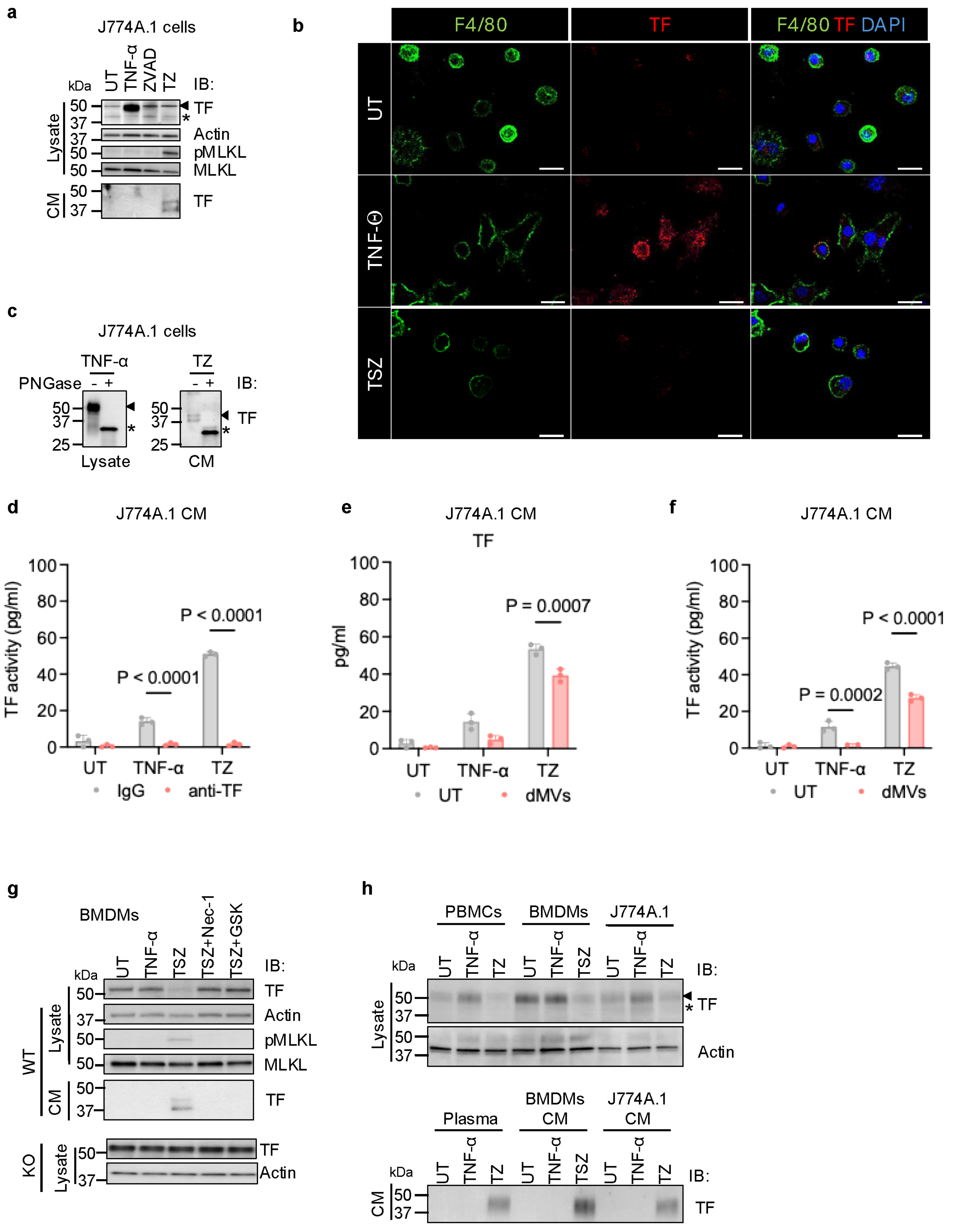

a
J774A.1 cells
kDa
UT
TNF-α
ZVAD
TZ
IB:
TF
Actin
pMLKL
MLKL
TF
Lysate
CM
50
37
b
F4/80
TF
F4/80 TF DAPI
UT
TNF-α
TSZ
c
J774A.1 cells
TNF-α
TZ
PNGase
IB:
TF
Lysate
CM
50
37
25
d
J774A.1 CM
TF activity (pg/ml)
P < 0.0001
P < 0.0001
UT
TNF-α
TZ
IgG
anti-TF
e
J774A.1 CM
TF
pg/ml
P = 0.0007
UT
TNF-α
TZ
UT
dMVs
f
J774A.1 CM
TF activity (pg/ml)
P < 0.0001
P = 0.0002
UT
TNF-α
TZ
UT
dMVs
g
BMDMs
kDa
UT
TNF-α
TSZ
TSZ+Nec-1
TSZ+GSK
IB:
TF
Actin
pMLKL
MLKL
TF
TF
Actin
WT
KO
Lysate
CM
Lysate
h
PBMCs
BMDMs
J774A.1
kDa
UT
TNF-α
TZ
UT
TNF-α
TSZ
UT
TNF-α
TZ
IB:
TF
Actin
Lysate
Plasma
BMDMs CM
J774A.1 CM
CM

Supplementary information, Fig S7. Necroptosis prompts the release of sTF from cultured monocytes/macrophages

a J774A.1 cells were treated with TNF-α, ZVAD, or TZ for 6 h and the cell lysate and conditioned medium (CM) was examined by WB with the indicated antibodies. MVs were removed from CM. Arrowhead: TF bands. Star: unspecific bands.

b J774A.1 cells were either untreated or treated with TNF-α or TZ for 6h. Representative confocal microscopy images of J774A.1 cells stained with antibodies against F4/80 (green) and TF (red). The nuclei were labeled with DAPI (blue). Scale bar=10μm.

c J774A.1 cells were treated with either TNF-α or TZ for 6h. Cell lysate and conditioned medium (CM) was collected and processed with PNGase. MVs were removed from CM. Samples were examined by WB analysis with the indicate antibodies. Arrowhead: glycoprotein. Star: de-glycosylated protein.

d TF was selectively depleted from the CM samples of UT, TNF-α, ZVAD, or TZ-treated J774A.1 cells using an antibody pull-down technique. The TF activities in the plasma pre-processed with either control IgG or anti-TF antibody was measured by PCA assay. n=3 per group.

e-f MVs were isolated from CM of UT, TNF-α or TZ-treated J774A.1 cells by spin down at 20,000xg for 20min. (E) TF level in J774A.1 CM and MVs-depleted CM was measured by ELISA. n=3 per group. dMVs: depleted microvesicles. (F) TF activity in treated J774A.1 CM and MVs-depleted CM was examined by PCA assay. n=3 per group. dMVs: depleted microvesicles.

g Bone marrow derived macrophages (BMDMs) were isolated from WT (upper panel) and MLKL KO (lower panel) mice and treated as indicated. Cells were pre-treated for 1h with necroptosis inhibitors, Necrostatin-1 (Nec-1) or GSK'872 (GSK), followed by TNF-α+Smac mimetic+ZVAD (TSZ) for 16h treatment. Cell lysate and CM were collected for WB with the indicated antibodies. MVs were removed from CM.

h Mouse PBMCs and plasma from untreated, TNF-α, or TZ treated WT mice were collected at 6h post treatment. WT BMDMs lysate and CM were collected 6h post untreated, TNF-α, or TSZ treatment. J774A.1 cell lysate and CM were collected 6h post untreated, TNF-α, or TZ treatment. MVs were removed from plasma and CM. Samples were examined by WB with the indicated antibodies. Arrowhead: TF bands. Star: unspecific bands.
