# Supplementary material for: Soluble tissue factor generated by necroptosis-triggered shedding is responsible for thrombosis
Source: Cell Res. 2025 Sep 12;35(11):840–58. doi: 10.1038/s41422-025-01167-8 (PMC12589612; doi:10.1038/s41422-025-01167-8)
Supplement: Supplementary file 8 — Fig. S8 [file 41422_2025_1167_MOESM8_ESM.pdf]

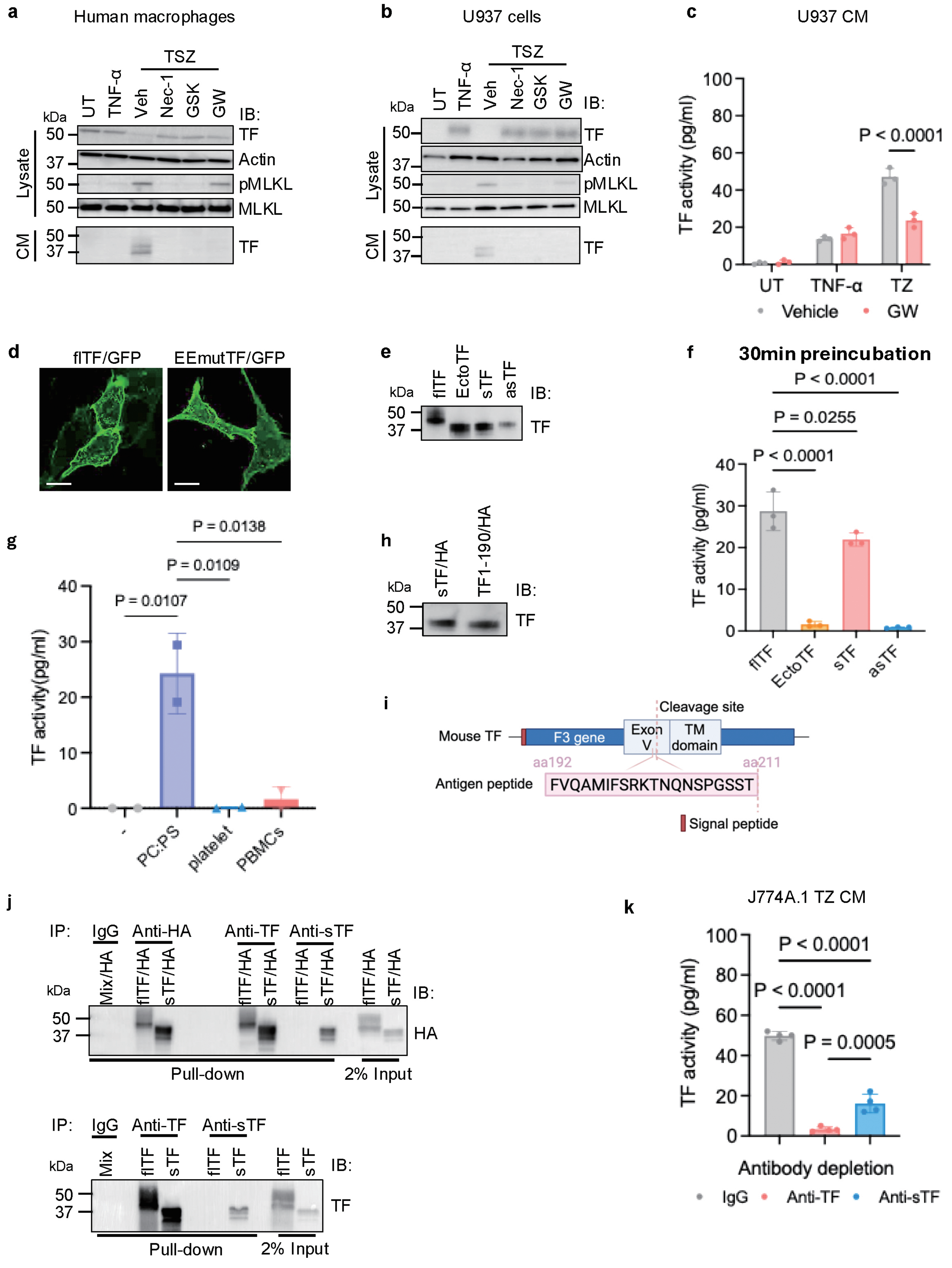
a
Human macrophages
TSZ
UT
TNF-α
Veh
Nec-1
GSK
GW
IB:
kDa
50
37
50
50
50
37
TF
Actin
pMLKL
MLKL
TF
Lysate
CM
b
U937 cells
TSZ
kDa
UT
TNF-α
Veh
Nec-1
GSK
GW
IB:
TF
Actin
pMLKL
MLKL
TF
Lysate
CM
c
U937 CM
TF activity (pg/ml)
100
80
60
40
20
0
P < 0.0001
UT
TNF-α
TZ
Vehicle
GW
d
flTF/GFP
EEmutTF/GFP
e
kDa
flTF
EctoTF
sTF
asTF
IB:
50
37
TF
f
30min preincubation
P < 0.0001
P = 0.0255
P < 0.0001
TF activity (pg/ml)
40
30
20
10
0
flTF
EctoTF
sTF
asTF
g
P = 0.0138
P = 0.0109
P = 0.0107
TF activity(pg/ml)
40
30
20
10
0
-
PC:PS
platelet
PBMCs
h
kDa
sTF/HA
TF1-190/HA
IB:
50
37
TF
i
Cleavage site
Mouse TF
F3 gene
Exon V
TM domain
aa192
aa211
Antigen peptide
FVQAMIFSRKTNQNSPGSST
Signal peptide
j
IP:
IgG
Anti-HA
Anti-TF
Anti-sTF
Mix/HA
flTF/HA
sTF/HA
flTF/HA
sTF/HA
flTF/HA
sTF/HA
flTF/HA
sTF/HA
IB:
kDa
50
37
HA
Pull-down
2% Input
IP:
IgG
Anti-TF
Anti-sTF
kDa
Mix
flTF
sTF
flTF
sTF
flTF
sTF
IB:
50
37
TF
Pull-down
2% Input
k
J774A.1 TZ CM
TF activity (pg/ml)
100
80
60
40
20
0
P < 0.0001
P < 0.0001
P = 0.0005
Antibody depletion
IgG
Anti-TF
Anti-sTF

Supplementary information, Fig S8. ADAMs cleavage in cultured macrophages

a Primary human macrophages were differentiated from elutriated monocytes. Cells were pre-treated with vehicle control, Nec-1, GSK, or GW for 1h followed by triggering necroptosis with TSZ treatment for 6h. Cell lysate and CM from treated human macrophages were collected for WB analysis with the indicated antibodies. MVs were removed from CM.

b-c U937 cells were pre-treated with vehicle control, Nec-1, GSK, or GW for 1h, followed by TNF-α or TSZ treatment for 6h. (B) Cell lysate and CM from untreated, TNF-α, and TZ treated U937 cells was examined by WB with the indicated antibodies. MVs were removed from CM. (C) CM from untreated, TNF-α, and TSZ treated U937 cells was examined for TF activity by PCA assay. n=3 per group.

d Representative confocal microscopy images of full-length TF (flTF) /GFP/HA or E211E212 mutated TF (EEmutTF)/GFP/HA transfected cells. Scale bar=10μm.

e-f Same amount of recombinant flTF, EctoTF, sTF, or asTF protein was purified from HEK293 cells as detected by WB. (F) These TF proteins (100pg/assay) were relipidated and incubated with human FVIIa (final concentration of 2.4nM) and human FX (Prolytix, HCX-0050, final concentration of 73.2nM) in an HBSA buffer supplemented with 5mM CaCl2 at 37°C for 30min, followed by PCA assay. n=3 per group.

g Recombinant mouse sTF protein was purified from HEK293 cells. This protein was incubated without or with PC:PS, platelets or PBMCs from untreated WT mouse blood and procoagulant activity was examined by PCA assay.

h Same amount of recombinant sTF, or TF1-190 protein was purified from HEK293 cells as detected by WB.

i The diagram of antigen peptide used for sTF polyclonal antibody generation.

j HA tagged (upper panel) and untagged (lower panel) and flTF or sTF plasmids was transfected into HEK293 cells for 24h. Lysate was collected and pulled down with IgG, anti-TF antibody, or anti-sTF antibody. Mix indicates the 1:1 mixture of flTF and sTF transfected HEK293 lysate. The pull-down product was analyzed by WB with indicated antibodies.

k TF was depleted from the CM samples of TZ-treated J774A.1 cells by pull-down using either control IgG, anti-TF antibody, or anti-sTF antibody. The TF activity was measured in TF depleted (post-pulldown) CM by PCA assay. n=4 per group.
