# Supplementary material for: Soluble tissue factor generated by necroptosis-triggered shedding is responsible for thrombosis
Source: Cell Res. 2025 Sep 12;35(11):840–58. doi: 10.1038/s41422-025-01167-8 (PMC12589612; doi:10.1038/s41422-025-01167-8)
Supplement: Supplementary file 9 — Fig. S9 [file 41422_2025_1167_MOESM9_ESM.pdf]

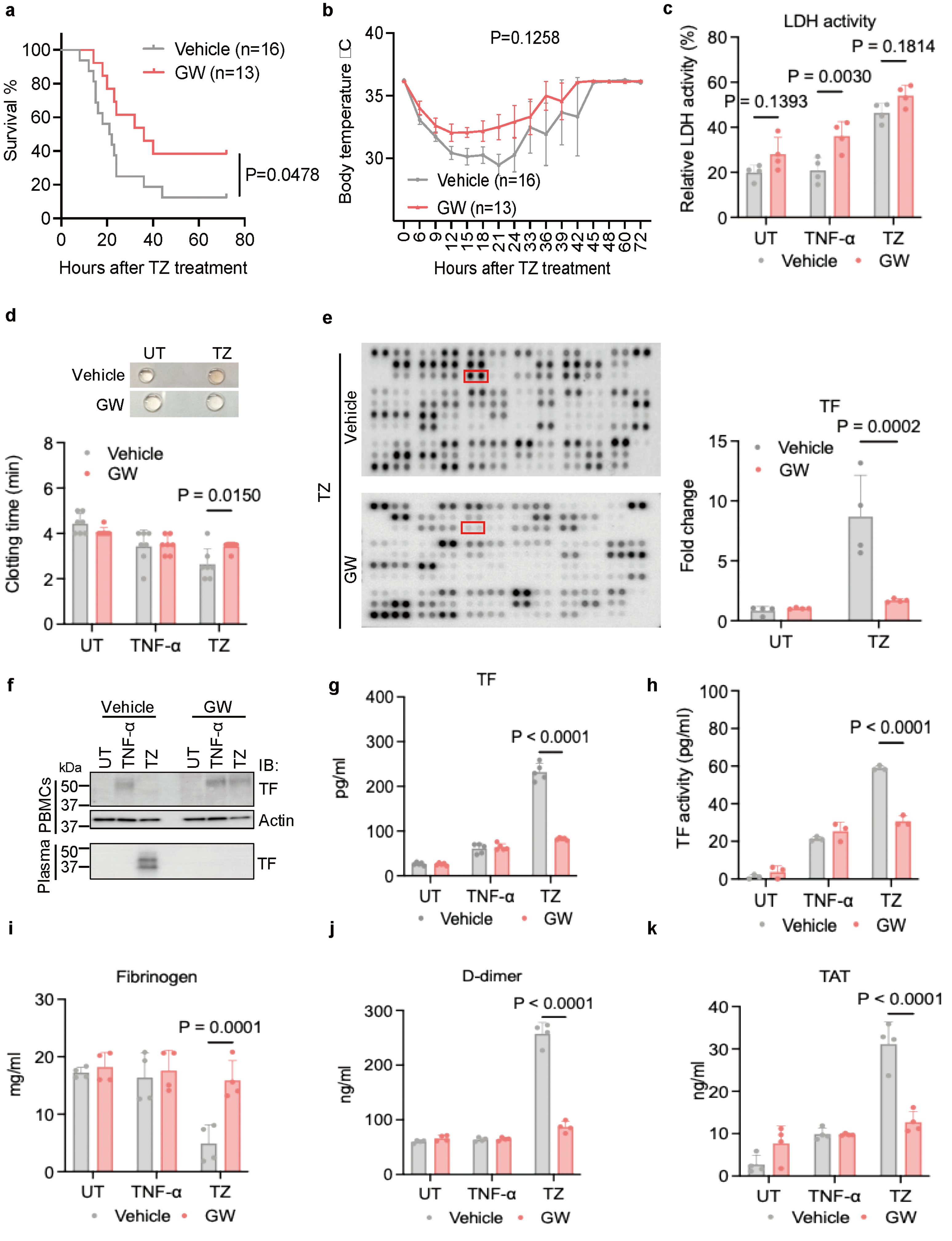

a
Survival %
Vehicle (n=16)
GW (n=13)
P=0.0478
Hours after TZ treatment
b
Body temperature □C
P=0.1258
Vehicle (n=16)
GW (n=13)
Hours after TZ treatment
c
LDH activity
Relative LDH activity (%)
P = 0.1393
P = 0.0030
P = 0.1814
UT
TNF-α
TZ
Vehicle
GW
d
UT
TZ
Vehicle
GW
Clotting time (min)
P = 0.0150
UT
TNF-α
TZ
e
TZ
Vehicle
GW
TF
Fold change
P = 0.0002
Vehicle
GW
UT
TZ
f
Vehicle
GW
UT
TNF-α
TZ
IB:
kDa
PBMCs
Plasma
TF
Actin
g
TF
pg/ml
P < 0.0001
UT
TNF-α
TZ
Vehicle
GW
h
TF activity (pg/ml)
P < 0.0001
UT
TNF-α
TZ
Vehicle
GW
i
Fibrinogen
mg/ml
P = 0.0001
UT
TNF-α
TZ
Vehicle
GW
j
D-dimer
ng/ml
P < 0.0001
UT
TNF-α
TZ
Vehicle
GW
k
TAT
ng/ml
P < 0.0001
UT
TNF-α
TZ
Vehicle
GW

Supplementary information, Fig S9. Suppressing shedding of flTF prevented TZ-induced hypercoagulation

WT mice were administered vehicle control or 100μg/kg GW 280264X (i.p.) at 24h and 48h prior to TNF-α or TZ challenge.

a The survival curve of vehicle or GW pre-treated mice during TZ-mediated inflammation.

b The body temperature plot of vehicle or GW pre-administered mice during TZ-mediated inflammation.

c LDH activity was measured in plasma samples from vehicle control and GW pre-treated mice at 6 h post TNF-α or TZ challenge. n=4 per group.

d Image of hemolysis as assessed by color change in plasma samples from vehicle control and GW pre-treated mice at 6h post TZ challenge. Representative images are shown in upper panel. Clotting time in plasma samples from vehicle control and GW pre-treated mice was measured in capillary tubes at 6 h post TNF-α or TZ challenge and shown in lower panel. n=7 per group.

e Plasma samples from vehicle control and GW pre-treated mice were examined with cytokine array at 6h post TZ. Representative arrays are shown here. TF spots were highlighted in red box. Quantification of TF expression on array was shown in right panel.

f PBMCs and plasma from mice pre-treated with either vehicle or GW and challenged with TNF-α alone or with TZ were examined by WB with the indicated antibodies. MVs were removed from plasma.

g The TF level in the plasma from mice pre-treated with either vehicle or GW and challenged with TNF-α alone or with TZ was measured by ELISA. n=5 per group.

h TF activity was measured in plasma samples from mice pre-treated with either vehicle or GW and challenged with TNF-α alone or with TZ by PCA assay. n=3 per group.

i-k Plasma samples were collected from vehicle control and GW pre-treated mice at 6h post TNF-α or TZ challenge. Levels of Fibrinogen **(i)**, D-dimer **(j)**, and TAT **(k)** were measured in plasma samples by ELISA. n=4 per group.
