# Supplementary material for: Soluble tissue factor generated by necroptosis-triggered shedding is responsible for thrombosis
Source: Cell Res. 2025 Sep 12;35(11):840–58. doi: 10.1038/s41422-025-01167-8 (PMC12589612; doi:10.1038/s41422-025-01167-8)
Supplement: Supplementary file 10 — Fig. S10 [file 41422_2025_1167_MOESM10_ESM.pdf]

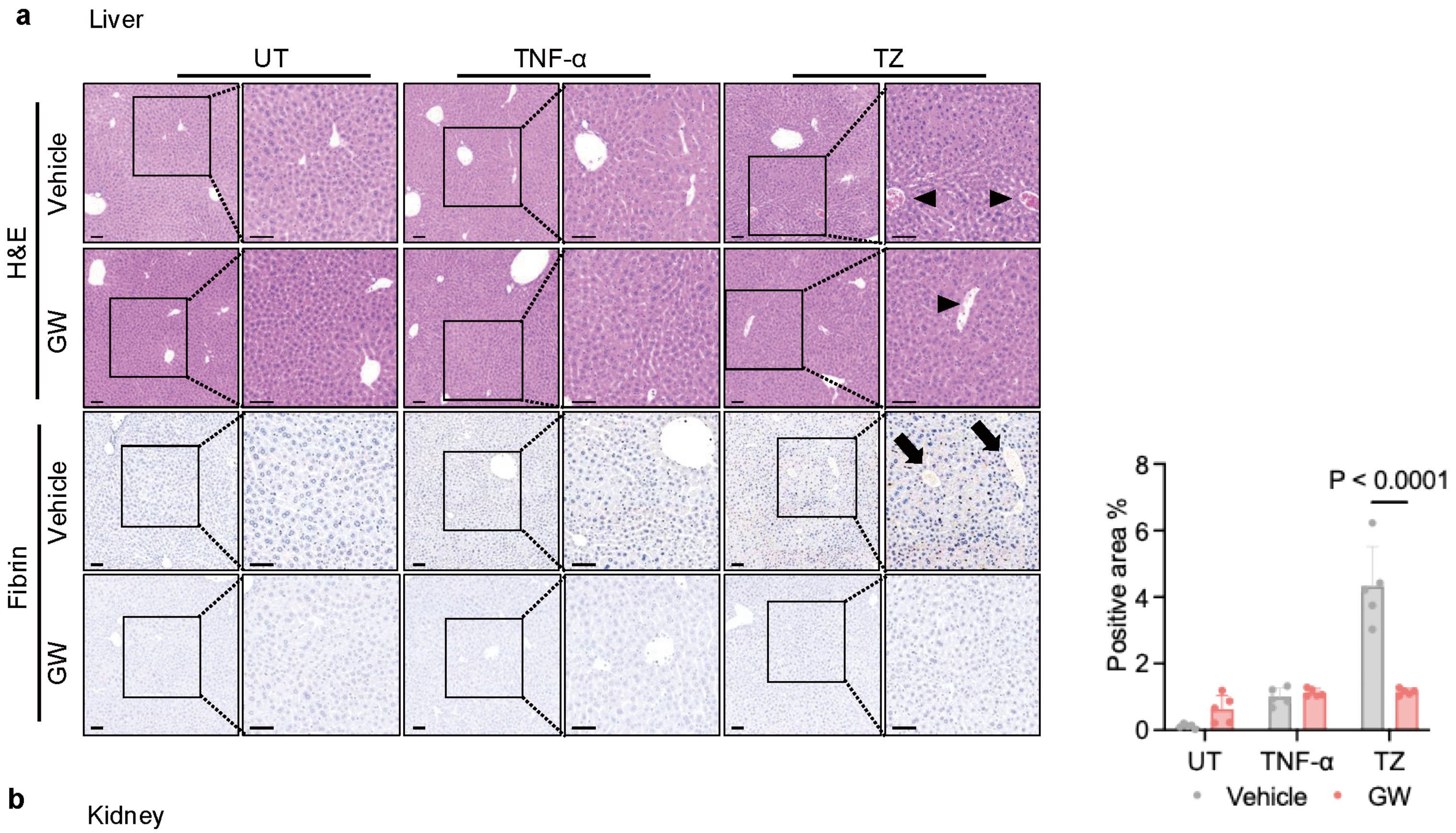

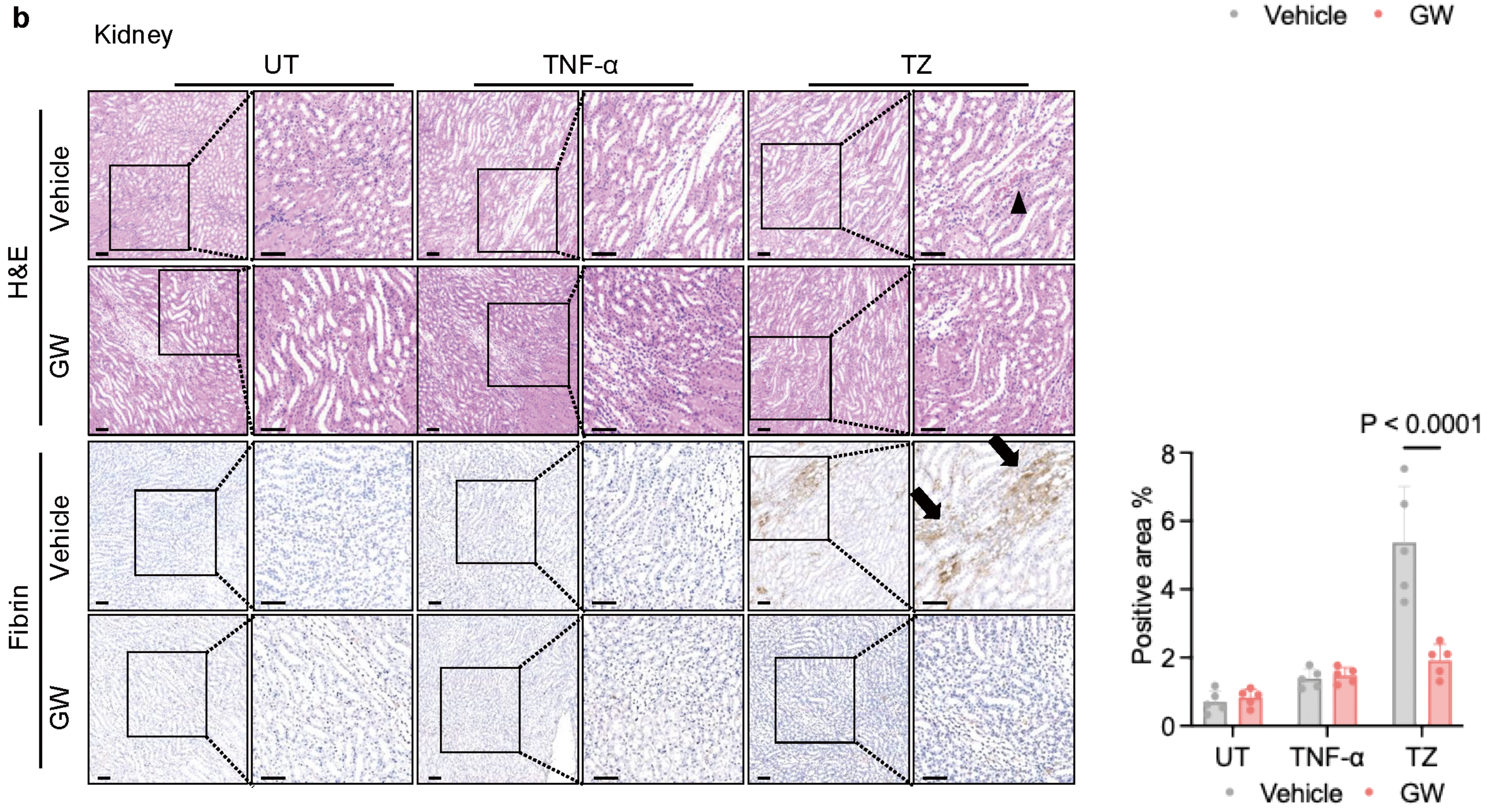

Supplementary information, Fig S10. GW prevented TZ-triggered thrombosis

a Liver sections from vehicle control and GW pre-treated mice were examined at 6h post TNF-α or TZ challenge. Representative images of H&E staining are shown in upper left panel. Representative images of fibrin IHC staining are shown in lower left panel. Fibrin IHC staining quantification is shown in right panel. Scale bar=40μm. Arrowhead: thrombus. Arrow: fibrin signal.

b Kidney sections from vehicle control and GW pre-treated mice were examined at 6h post TNF-α or TZ challenge. Representative images of H&E staining are shown in upper left panel. Representative images of fibrin IHC staining are shown in lower left panel. Fibrin IHC staining quantification is shown in right panel. Scale bar=40μm. Arrowhead: thrombus. Arrow: fibrin signal.
