# Supplementary material for: Soluble tissue factor generated by necroptosis-triggered shedding is responsible for thrombosis
Source: Cell Res. 2025 Sep 12;35(11):840–58. doi: 10.1038/s41422-025-01167-8 (PMC12589612; doi:10.1038/s41422-025-01167-8)
Supplement: Supplementary file 11 — Fig. S11 [file 41422_2025_1167_MOESM11_ESM.pdf]

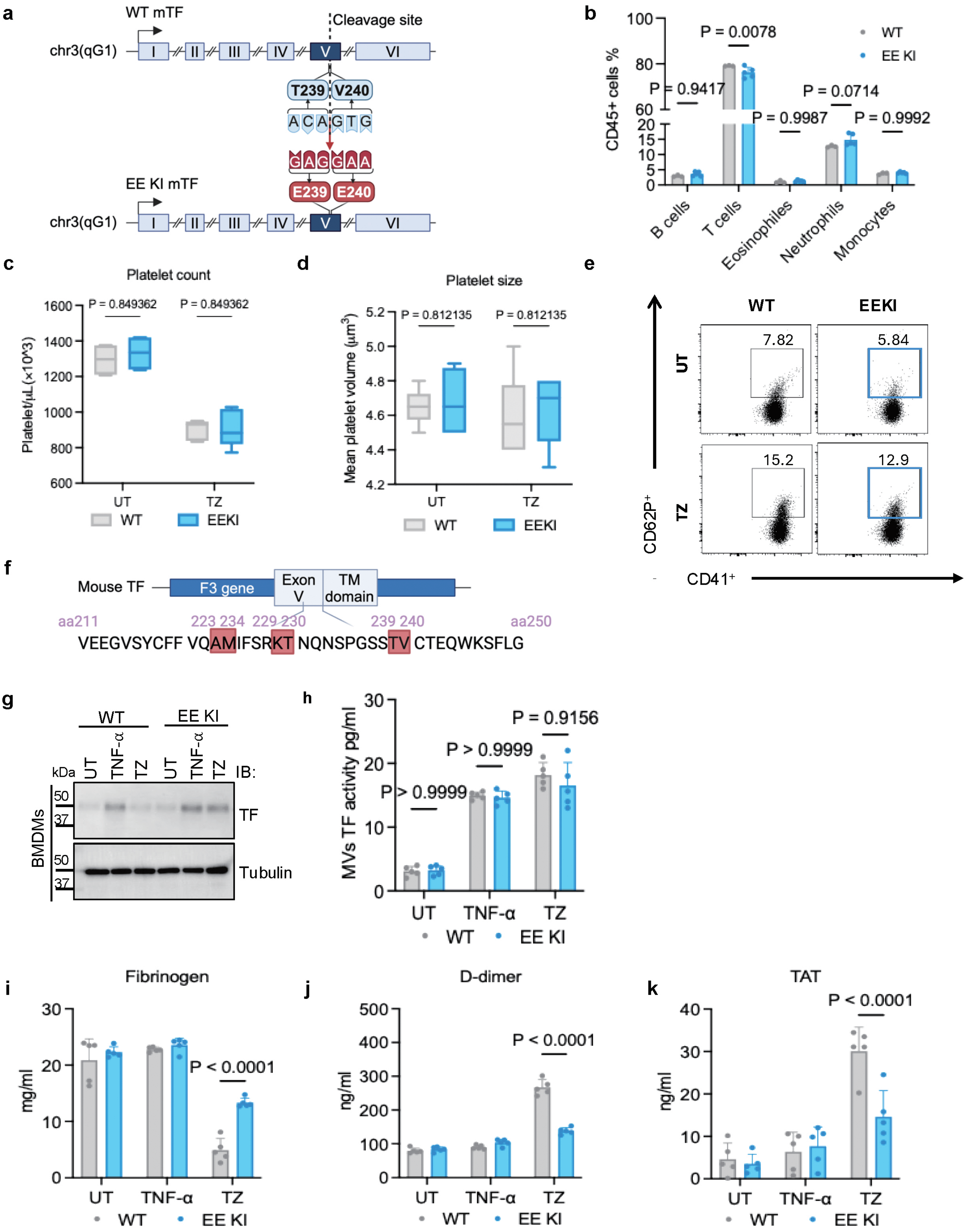

a
WT mTF
Cleavage site
chr3(qG1)
I II III IV V VI
T239 V240
A C A G T G
G A G G A A
E239 E240
EE KI mTF
chr3(qG1)
I II III IV V VI
b
CD45+ cells %
P = 0.0078
WT
EE KI
P = 0.9417
P = 0.0714
P = 0.9987
P = 0.9992
B cells
T cells
Eosinophiles
Neutrophils
Monocytes
c
Platelet count
P = 0.849362
P = 0.849362
Platelet/μL (×10^3)
UT
TZ
WT
EEKI
d
Platelet size
P = 0.812135
P = 0.812135
Mean platelet volume (μm³)
UT
TZ
WT
EEKI
e
WT
EEKI
UT
TZ
7.82
5.84
15.2
12.9
CD62P⁺
CD41⁺
f
Mouse TF
F3 gene
Exon V
TM domain
aa211
223 234
229 230
239 240
aa250
VEEGVSYCFF VQAMIFSRKT NQNSPGSSTV CTEQWKSFLG
g
WT
EE KI
UT TNF-α TZ UT TNF-α TZ
kDa
IB:
BMDMs
50
37
TF
50
37
Tubulin
h
MVs TF activity pg/ml
P = 0.9156
P > 0.9999
P > 0.9999
UT
TNF-α
TZ
WT
EE KI
i
Fibrinogen
mg/ml
P < 0.0001
UT
TNF-α
TZ
WT
EE KI
j
D-dimer
ng/ml
P < 0.0001
UT
TNF-α
TZ
WT
EE KI
k
TAT
ng/ml
P < 0.0001
UT
TNF-α
TZ
WT
EE KI

Supplementary information, Fig S11. TF EE KI mice were resistant from TZ induced sTF shedding or thrombosis

a The diagram illustrates the genomic mutation sites in mouse TF EE knock-in mice (EE KI). The point mutations located at exon 5 (239T to E, 240V to E) of the mouse TF gene, were introduced using CRISPR–Cas9 technology.

b Whole peripheral blood was collected from untreated WT and EE KI mice. The leukocytes were labeled with fluorescent antibodies and analyzed with flow cytometry (details in Methods). n=3 in WT groups; n=5 in EE KI groups.

c-e Whole peripheral blood was collected from untreated or TZ-challenged WT or EE KI mice at 16h after treatment to assess platelet counts (**c**), platelet size (**d**), and platelet activation analysis (**e**).

f Diagram showed the three predicted ADAM10/17 cleavage sites in mouse TF by MEROPS. The predicted cleavage sites were highlighted in red boxes. The major cleavage site resided in T211V212.

g Mouse BMDMs were generated by obtaining bone marrow cells from WT or EE KI mice and culturing in complete DMEM supplemented with mouse M-CSF. After 7 days, BMDMs were untreated or treated with TNF-a or TZ for 6 h and then cell lysates were examined by WB with the indicated antibodies.

h MVs were isolated in plasma from untreated, TNF-α, or TZ-challenged WT and EE KI mice at 6h post treatment. TF activity of isolated MVs were measured using an PCA assay. n=5 per group.

i-k Plasma levels of Fibrinogen, D-dimer, and TAT in WT or EE KI mice untreated or challenged with TNF-α alone or with TZ were examined by ELISA assay. n=5 per group.
