# Supplementary material for: Soluble tissue factor generated by necroptosis-triggered shedding is responsible for thrombosis
Source: Cell Res. 2025 Sep 12;35(11):840–58. doi: 10.1038/s41422-025-01167-8 (PMC12589612; doi:10.1038/s41422-025-01167-8)
Supplement: Supplementary file 12 — Fig. S12 [file 41422_2025_1167_MOESM12_ESM.pdf]

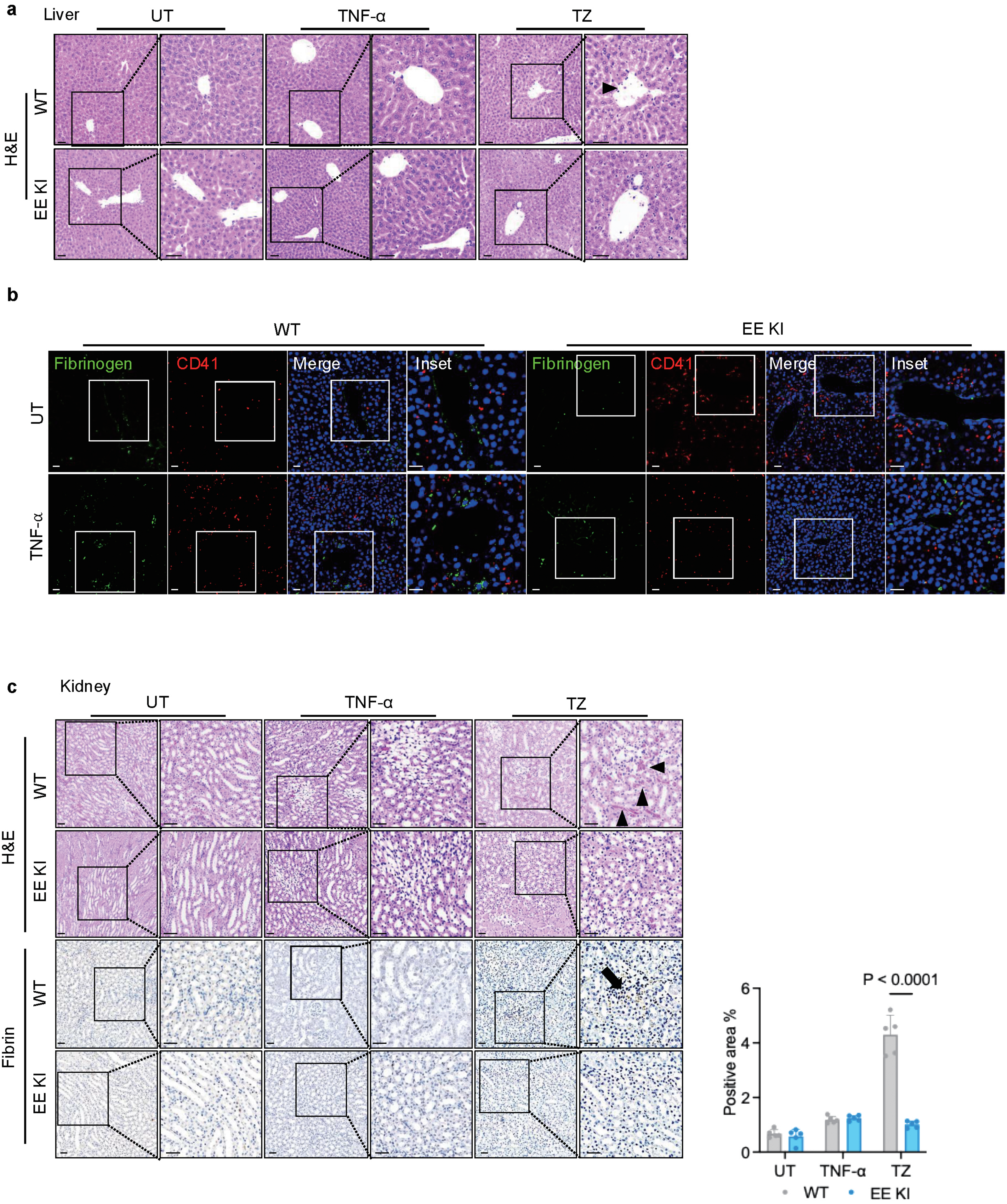
a
Liver
UT
TNF-α
TZ
H&E
WT
EE KI
b
WT
EE KI
Fibrinogen
CD41
Merge
Inset
UT
TNF-α
c
Kidney
UT
TNF-α
TZ
H&E
WT
EE KI
Fibrin
WT
EE KI
P < 0.0001
Positive area %
6
4
2
0
UT
TNF-α
TZ
WT
EE KI

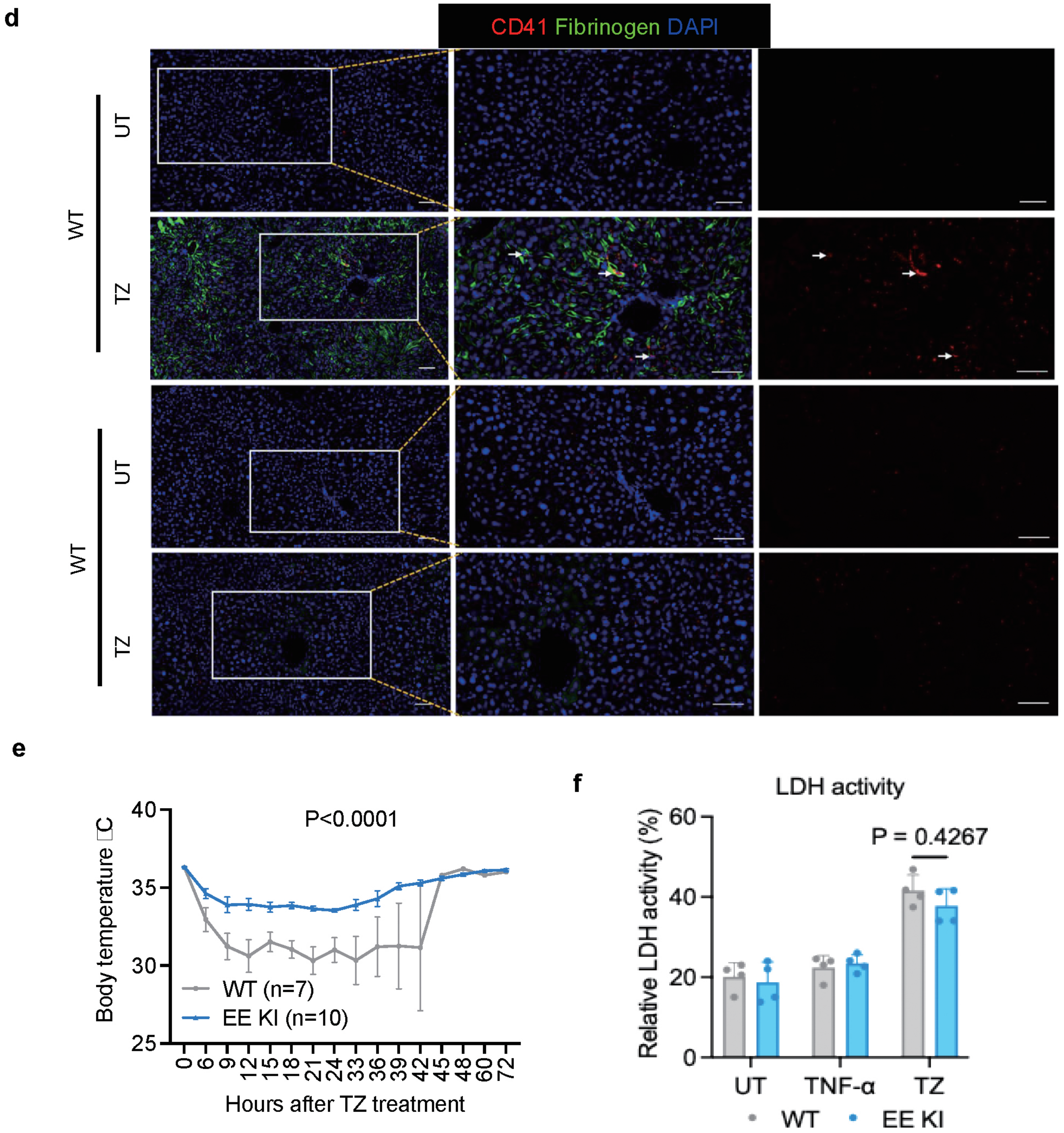

Supplementary information, Fig S12. TF EE KI mice were resistant from TZ induced thrombosis

a Liver sections from WT and EE KI mice without treatment or at 6h post TNF-α or TZ challenge. Representative images of H&E staining. Scale bar=40μm. Arrowhead: thrombus.

b Representative images of IF staining of liver sections from WT and EE KI mice without treatment or challenged with TNF-α for 6h. Fibrinogen deposition was indicated by a green signal, platelets were labeled with CD41 (red), and nuclei were stained blue. Scale bar=40μm.

c Kidney sections from WT and EE KI mice without treatment or at 6h post TNF-α or TZ challenge. Representative images of H&E staining are shown in upper left panel. Representative images of fibrin IHC staining are shown in lower left panel. Fibrin IHC staining quantification is shown in right panel. Scale bar=40μm. Arrowhead: thrombus. Arrow: fibrin signal.

d Representative immunofluorescence (IF) images of liver sections from untreated or TZ challenged WT/EEKI mice at 16 h post treatment. Fibrinogen deposition was shown in green, platelets were labeled with CD41 (red), and nuclei were stained blue. Scale bar=50μm. Arrows indicate aggregation of platelets in fibrinogen accumulated sinusoids.

e Body temperature change in WT and EE KI mice after TZ challenge.

f LDH activity was measured in plasma from untreated, TNF-α, or TZ-challenged WT and EE KI mice at 6h post treatment. n=4 per group.
