# Supplementary material for: Soluble tissue factor generated by necroptosis-triggered shedding is responsible for thrombosis
Source: Cell Res. 2025 Sep 12;35(11):840–58. doi: 10.1038/s41422-025-01167-8 (PMC12589612; doi:10.1038/s41422-025-01167-8)
Supplement: Supplementary file 13 — Fig. S13 [file 41422_2025_1167_MOESM13_ESM.pdf]

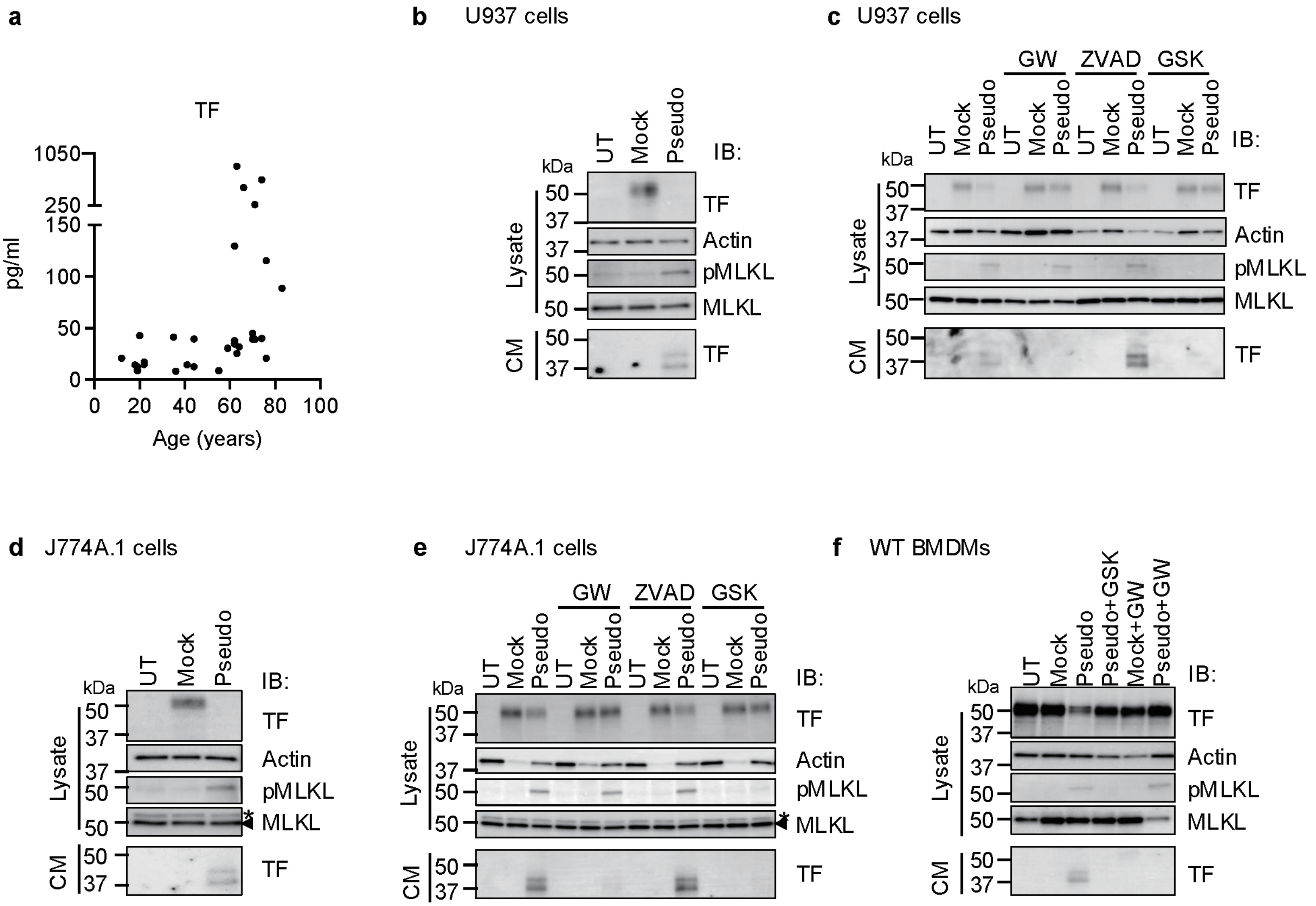

Supplementary information, Fig S13. SARS-CoV-2 infection stimulates sTF production

a Distribution of TF level in plasma samples from either normal donors (n=5) or COVID-19 patients (n=30) by age.

b-f U937 cells (**b,c**), J774A.1 cells (**d,e**), and WT BMDMs (**f**) were infected with mock virus or SARS-CoV-2 pseudo virus for 60h. Inhibitors (GW, ZVAD, or GSK) were added 1h before the onset of infection and replenished every 24h. Lysate and CM of infected cells were analyzed by WB with the indicated antibodies. MVs were removed from CM. Arrowhead: MLKL bands. Star: unspecific bands.
