# Supplementary material for: Soluble tissue factor generated by necroptosis-triggered shedding is responsible for thrombosis
Source: Cell Res. 2025 Sep 12;35(11):840–58. doi: 10.1038/s41422-025-01167-8 (PMC12589612; doi:10.1038/s41422-025-01167-8)
Supplement: Supplementary file 14 — Fig. S14 [file 41422_2025_1167_MOESM14_ESM.pdf]

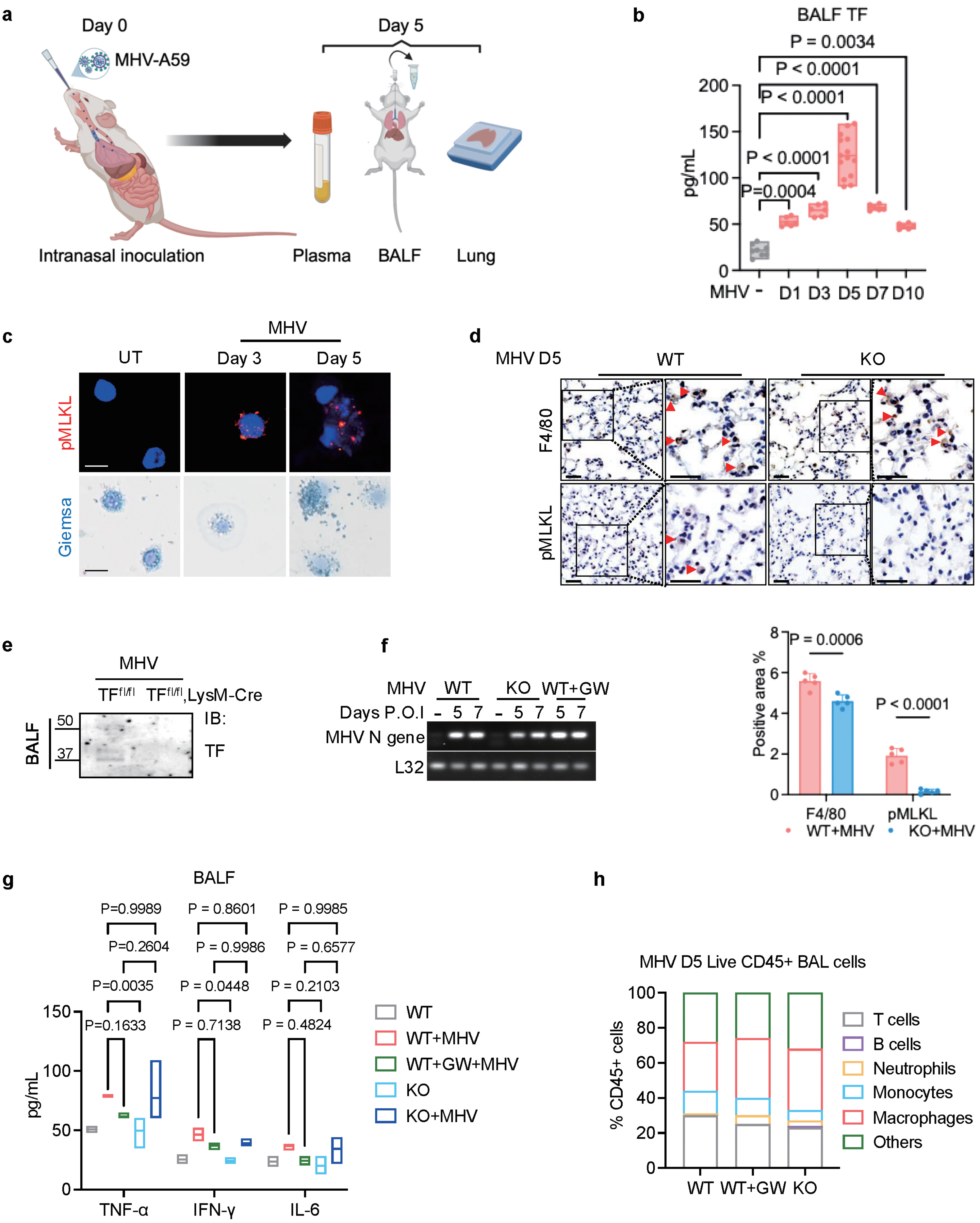

a
Day 0
MHV-A59
Day 5
Intranasal inoculation
Plasma
BALF
Lung
b
BALF TF
P = 0.0034
P < 0.0001
P < 0.0001
P < 0.0001
P=0.0004
pg/mL
200
150
100
50
0
MHV -
D1 D3 D5 D7 D10
c
MHV
UT
Day 3
Day 5
pMLKL
Giemsa
d
MHV D5
WT
KO
F4/80
pMLKL
e
MHV
TF$^{fl/fl}$
TF$^{fl/fl}$,LysM-Cre
IB:
TF
BALF
50
37
f
MHV WT KO WT+GW
Days P.O.I - 5 7 - 5 7 5 7
MHV N gene
L32
P = 0.0006
P < 0.0001
Positive area %
8
6
4
2
0
F4/80
pMLKL
WT+MHV
KO+MHV
g
BALF
P=0.9989
P = 0.8601
P = 0.9985
P=0.2604
P = 0.9986
P = 0.6577
P=0.0035
P = 0.0448
P = 0.2103
P=0.1633
P = 0.7138
P = 0.4824
pg/mL
150
100
50
0
TNF-α
IFN-γ
IL-6
WT
WT+MHV
WT+GW+MHV
KO
KO+MHV
h
MHV D5 Live CD45+ BAL cells
% CD45+ cells
100
80
60
40
20
0
WT
WT+GW
KO
T cells
B cells
Neutrophils
Monocytes
Macrophages
Others

Supplementary information, Fig S14. Mouse MHV-A59 infection model

a Scheme of mouse MHV-A59 (MHV) model. FVB/J WT or MLKL KO mice were intranasally inoculated with $15x10^4$ PFU MHV. Samples are collected on Day 5 post infection unless otherwise specified.

b Bronchoalveolar lavage fluid (BALF) was collected at different time points post MHV inoculation in WT mice. TF levels in BALF was measured by ELISA. n=8 in uninfected group, n=6 in Day1, 3, 7, 10 post MHV inoculation group, n=14 in Day 5 post MHV inoculation group

c Bronchoalveolar lavage (BAL) cells were collected from uninfected WT mice or on Day 3 and Day 5 post MHV inoculation. Representative confocal microscopy images of pMLKL (red) staining are shown in upper panel. Nuclei was labeled with DAPI (blue). Representative images of Giemsa staining of BAL cells are shown in lower panel. Scale bar=10µm.

d Lung sections from uninfected or MHV inoculated WT, and MLKL KO mice were examined. Representative images of F4/80 and pMLKL IHC staining are shown. The quantification of F4/80 and pMLKL IHC signals are shown in lower panel. Arrowhead: IHC positive signal.

e Bronchoalveolar lavage fluid (BALF) was collected at 5 days post MHV inoculation in $TF^{fl/fl}$ and $TF^{fl/fl}$,LysM-Cre mice. Soluble TF level in BALF was analyzed by WB with indicated antibody.

f-h WT mice were administered with vehicle control or 100µg/kg GW (i.p.) immediately prior to the MHV intranasal inoculation, 2 days post-inoculation, and again 4 days post-inoculation.

(**f**) Lung samples from uninfected or MHV inoculated WT, GW pre-administered, and MLKL KO mice were examined. MHV N gene was quantified in lung samples on Day 5 and Day 7 post MHV inoculation by RT-PCR.

(**g**) BALF samples from uninfected or MHV inoculated WT and MLKL KO mice were examined on Day 5 post MHV inoculation by mouse CBA assay. The quantification of cytokines was calculated with BD CBA program. n=3 per group.

(**h**) BAL cells from uninfected or MHV inoculated WT, GW pre-administered, and MLKL KO mice on Day 5 post MHV inoculation was analyzed by flow cytometry. The composition of CD45 positive BAL cells was evaluated by specific markers (details in Methods). n=3 pergroup.
