# Supplementary material for: Soluble tissue factor generated by necroptosis-triggered shedding is responsible for thrombosis
Source: Cell Res. 2025 Sep 12;35(11):840–58. doi: 10.1038/s41422-025-01167-8 (PMC12589612; doi:10.1038/s41422-025-01167-8)
Supplement: Supplementary file 15 — Fig. S15 [file 41422_2025_1167_MOESM15_ESM.pdf]

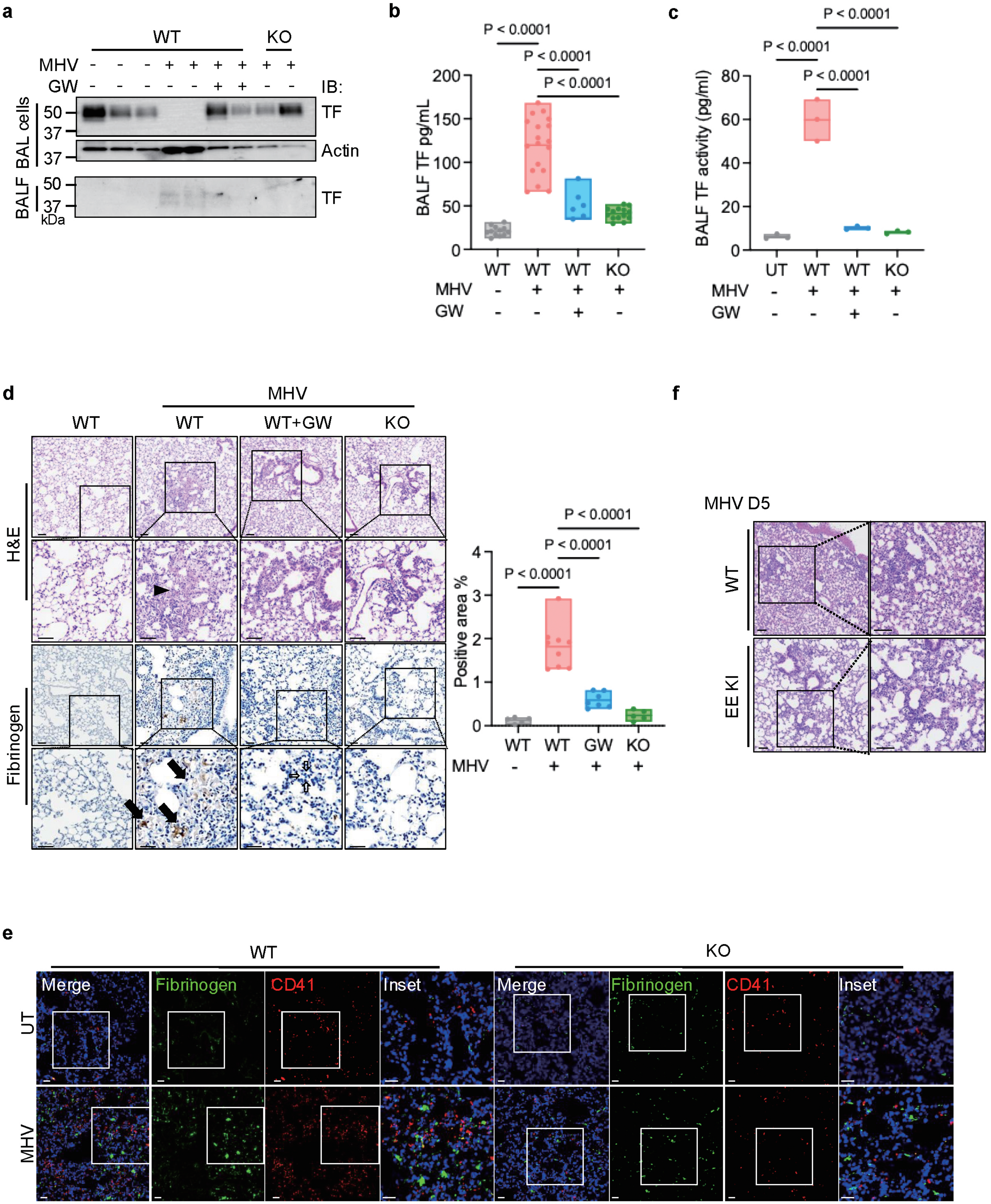
a
WT
KO
MHV
GW
IB:
BAL cells
BALF
50
37
kDa
TF
Actin
b
P < 0.0001
BALF TF pg/mL
MHV
GW
c
BALF TF activity (pg/ml)
UT
d
MHV
WT
WT+GW
KO
H&E
Fibrinogen
Positive area %
f
MHV D5
EE KI
e
Merge
Fibrinogen
CD41
Inset
UT

Supplementary information, Fig S15. Inhibition of necroptosis or TF cleavage alleviates thrombosis in viral infection

a-e WT and MLKL KO mice were intranasally inoculated with $15x10^4$ PFU MHV. WT mice were administered with vehicle control or 100μg/kg GW (i.p.) immediately prior to the MHV-A59 (MHV) intranasal inoculation, 2 days post-inoculation, and again 4 days post-inoculation. Analyses were conducted on day 5 post-infection.

(**a**) BALF and BAL cells from uninfected or WT, GW pre-administered, and MLKL KO mice post MHV infection were examined by WB with the indicated antibodies. MVs were removed from BALF samples.

(**b**) The TF level was measured in BALF from uninfected or WT, GW pre-administered, and MLKL KO mice post MHV infection by ELISA. n=14 in WT uninfected group. n=18 in WT MHV infected group. n=6 in GW pretreated MHV infected group. n=14 in MLKL KO MHV infected group.

(**c**) TF activity was examined in BALF from uninfected, or WT, GW pre-administered, and MLKL KO mice post MHV inoculation by PCA assay. n=3 per group.

(**d**) Representative images of uninfected or MHV exposed lung sections from WT, GW pre-treated, and MLKL KO mice. H&E staining was shown in upper left panel. Fibrinogen IHC staining was shown in lower left panel. Fibrinogen IHC staining quantification is shown in right panel. Scale bar=40μm. Arrowhead: thrombus. Arrow: fibrinogen signal.

(**e**) Representative images of IF staining of lung sections from uninfected or MHV inoculated WT and EE KI mice on Day 5 post MHV inoculation. Fibrinogen deposition was indicated by a green signal, platelets were labeled with CD41 (red), and nuclei were stained blue. Scale bar=40 μm.

(**f**) MHV exposed lung sections from WT and EE KI mice were examined. Representative images of H&E staining were shown. Scale bar=40μm.
