# Supplementary material for: Soluble tissue factor generated by necroptosis-triggered shedding is responsible for thrombosis
Source: Cell Res. 2025 Sep 12;35(11):840–58. doi: 10.1038/s41422-025-01167-8 (PMC12589612; doi:10.1038/s41422-025-01167-8)
Supplement: Supplementary file 16 — Fig. S16 [file 41422_2025_1167_MOESM16_ESM.pdf]

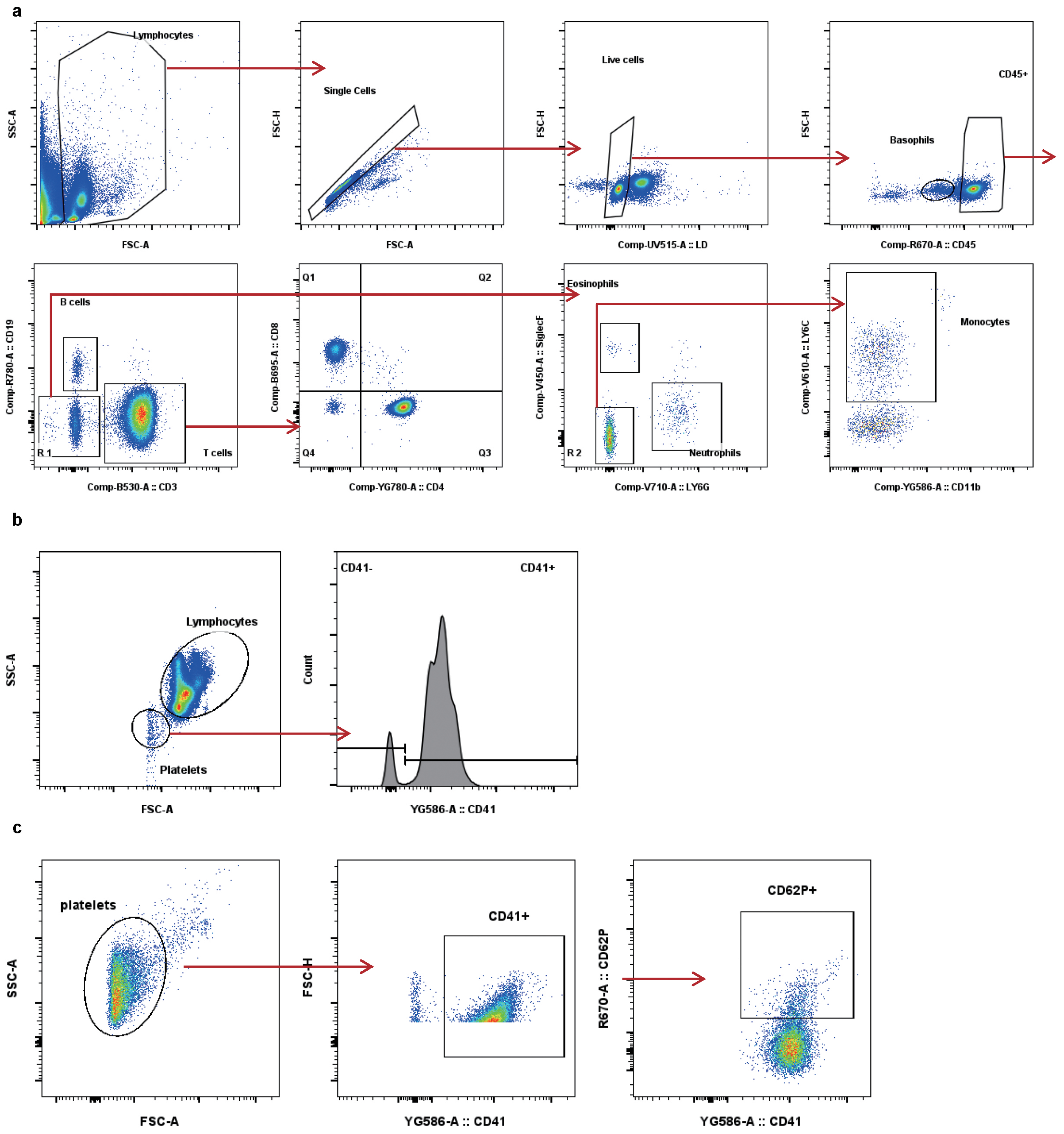

Supplementary information, Fig. S16 Gating strategy for PBMC and Platelets

a-c Gating strategy for (**a**) PBMC and (**b**) Platelets isolated from mouse whole blood to examine immune cells and platelets. Gating strategy for the percentage of activated platelets in platelet rich plasma (**c**).
